# The manufacture of the Baskerville typographic punches: the versatile *chaîne opératoire* of an 18^th^-century printing workshop

**DOI:** 10.1038/s40494-026-02504-9

**Published:** 2026-04-14

**Authors:** Julia Montes-Landa, Mark Box, Caroline Archer-Parré, Ann-Marie Carey, Maciej Pawlikowski, Marcos Martinón-Torres

**Affiliations:** 1 McDonald Institute for Archaeological Research, University of Cambridge, Cambridge, UK; 2 Department of Prehistory and Archaeology, University of Granada, Granada, Spain; 3 Cambridge Heritage Imaging Laboratory, Cambridge University Library, Cambridge, UK; 4 Centre for Printing History & Culture, Birmingham City University, Birmingham, UK; 5 School of Jewellery, Birmingham City University, Birmingham, UK; 6 Department of Archaeology, University of Cambridge, Cambridge, UK

## Abstract

The ‘material turn’ within the humanities has provided new possibilities for investigating historical technologies, and the *chaîne opératoire* offers a powerful framework for such research. Taking the history of printing as research arena, we present the first science-based study of typographic punches, focusing on the 18^th^-century collection manufactured by John Baskerville in Birmingham. By combining typology, microscopy, µCT, radiography and FTIR, with craftspeople's knowledge and historical sources, we reverse engineer the *chaîne opératoire* deployed to produce these tools. We characterise the technological tradition followed in Baskerville’s workshop and compare it to 19^th^/20^th^-century punch-making. Baskerville’s workshop was very versatile, and technological choices took into account performance factors and high craftsmanship standards. Many of the recorded choices are not described in 16^th^- and 17^th^-century punch-making texts. This reference study presents a chapter of the history of printing previously unexplored through material culture, and provides the method and theoretical framework for others.

## Introduction

Printing is a complex craft that intertwines and recombines existing technologies in novel ways, turning printing workshops into hubs for innovation. This complexity makes the study of printing technology challenging, but the study of surviving material culture can offer a powerful complementary approach to the topic. The ‘material culture turn’ has gained prominence in historical studies as it contributes insight into past technologies that written records alone cannot provide^1^. It shifts the focus to how artefacts were made, used and/or moved within or across socio-economic and technological systems, using the artefacts themselves as the main sources of information.

**Fig. 1:**
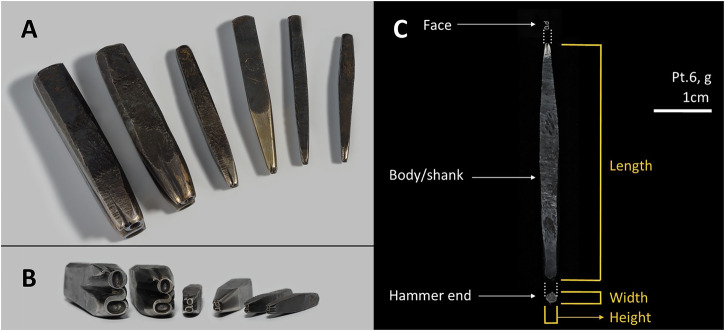
Examples of Baskerville punches. **A**, **B** General view of some of the punches of the collection. **C** Parts of a typographic punch.

Within this framework, this paper focuses on the typographic punches made at the workshop of John Baskerville (1707–75), 18^th^-century England’s foremost typefounder and printer. During his time, these small iron or steel (henceforth iron) rods with a character cut in relief and in reverse at one end (Fig. 1) were the first crafted tools necessary to produce books and any other printed matter. To print a text, individual punches for every capital and lowercase, roman and italic letter, number, ligature and symbol were produced by hand, usually in several different point sizes. These punches were then struck into a copper matrix to leave a negative right-reading imprint. After that, the copper matrix was introduced into a mould in which an alloy of antimony, lead and tin was poured to cast the printing type. The individual types were then arranged in a composing stick to form words and sentences. Then, they were taken to the printing press to be inked and impressed into paper to produce a book page. Thus, producing a single book using a specific typeface required manufacturing thousands of typographic punches to accommodate all the characters needed.

Although punches were at the core of early printing technology, little is known about their actual manufacture. Making a punch required craftsmanship at the crossroads between blacksmithing, metal engraving and typeface designing, but we lack understanding of how these technologies were recombined into a craft of its own: punch-making. Furthermore, the few available historical accounts of punch-making have not been confronted with the evidence of tool marks on the extant finished objects. This gap is aggravated by the fact that few historical collections of typographic punches have survived, the Baskerville collection being one of the largest, most complete and historically important. Currently hosted at the Cambridge University Library, it comprises 3240 items, most of them crafted at the workshop in Birmingham, and others added later during the 19^th^ and 20^th^ centuries across Europe. This collection is housed alongside some of the books for which it was used, another highly unusual fact.

To address this knowledge gap in a core chapter of the history of printing technology, we used an analytical approach widely employed in archaeological studies of technology but less so in studies of modern crafts: the *chaîne opératoire*. By combining a range of non-destructive scientific techniques and practice-based research, this paper reverse engineers 18^th^-century punch-making, characterises the Birmingham workshop’s technological tradition, and identifies differences in the ways of producing punches between the 18^th^ and the 19^th^–20^th^ centuries. As punch-making is on the red list of endangered crafts, this research is a novel contribution to the preservation of this knowledge, and offers an alternative view to the few contemporaneous records on this craft. It also opens the path to further studies on other collections by following this methodology.

To better understand these typographic punches, it is necessary to properly introduce John Baskerville (1707–75) and his crafted items. He was a writing master, a carver of gravestones, japanner, type founder, printer, industrialist, free thinker and Enlightenment figure. He was also the designer of the typeface that now bears his name, one which made Birmingham the centre of 18^th^-century printing and changed the course of type design^2^. Baskerville not only created one of the world’s most historically important typefaces, he also experimented with casting and setting type, improved the construction of the printing press, developed a new kind of paper and refined the quality of printing inks. His typographic experiments put him ahead of his time, had an international impact and did much to enhance the printing and publishing industries of his day^3^. Over the course of five years, despite having no training in the typographic arts (i.e. he was an ‘amateur’ in the full meaning of the world), Baskerville made his own punches in his Birmingham workshop with the assistance of the engraver John Handy (d. 1792), his workshop foreman William Martin (fl. 1786–1815) and the master printer Myles Swinney (fl. 1770–1812). In the absence of Baskerville’s workshop manual or examples of his printing types, the punches are of great importance as they are the only extant evidence of his working practices and rare survivors of the 18^th^-century typographic trade.

Baskerville’s punches were the tools he used to manufacture the printing types which were used on more than fifty volumes, from an edition of Virgil’s poetry (1757) to William Hunter’s *Anatomy of the Human Gravid Uterus* (1774). In 1758 Baskerville was appointed printer to the University of Cambridge, from where he issued multiple editions of the *Book of Common Prayer* and a folio edition Bible which is still regarded as one of the world’s most beautifully printed volumes.

When Baskerville died his type-founding equipment was sold in 1779 by his widow, Sarah Baskerville (fl. 1704–88), to Caron de Beaumarchais (1732–99), diplomat, publisher and playwright. Beaumarchais greatly admired Baskerville’s types and used them to produce the complete banned works of Voltaire from his printing house in Kehl in Germany. Claude Jacobe, a young French armourer, was engaged to inspect and correct, if necessary, all the punches cut in Birmingham, to add the diacritical characters required by the French, and to redesign some of the other characters^4^. To do this Jacobe was sent to England for two years to work under the supervision of John Handy and to learn the typographic techniques practiced in the Birmingham workshop^5^.

In 1790, with the printing of Voltaire complete, the punches were moved to Paris, where they remained for 150 years, during which time the punches changed ownership seven times, and their origin was forgotten. In 1917, the punches were in the possession of Fonderie Bertrand, which recast Baskerville’s fonts under the name of *Elzevirs ancient*. In November 1936, Bertrand’s set was sold to Fonderie Deberny et Peignot who recognised the Baskerville punches for what they were and commissioned freelance punch-cutters Charles Plumet and his former apprentice Charles Malin (1883–1955) to cut some replacement punches for those that were damaged or missing. In 1953, Charles Peignot (1897–1983), director of Deberny et Peignot, was persuaded to return the punches to Britain and presented them to the University of Cambridge on 12 March 1953 at a ceremony at Emmanuel College.

Considering that Baskerville started designing his typeface in around 1750 and died in 1775, it is necessary to address the contemporaneous literature on punch-making, highlighting the most important titles. One of the first early descriptions of punch-making was *Mechanick Exercises*, published in English in 1683 by Joseph Moxon^6^. He was a printer, mathematical lexicographer and hydrographer to Charles II. This pioneering work was soon followed by *Descriptions des Arts et Métiers* which was commissioned by the *Académie Royale* and contained sections on punch- and type-making. Despite being a 1690s initiative, the work only materialised in 1704 in French by Jacques Jaugeon^7^, a scholar and royal typographer attached to the service of Louis XIV, King of France. The fact that this is a manuscript makes accessibility very difficult for the average reader. James Mosley, a widely recognised historian of the history of printing, has published the plates related to punch- and type-making in English^8^, and provided transcriptions and translations of selected sections of Jaugeon’s text^9^, although the latter not related to punch-making but type-making.

In 1740, the anonymous *Kurtze, doch nützliche Anleitung von Form- und Stahl-Schneiden* (*Short, but useful guide to wood- and steel-cutting*) was published, which remains only available in German^10^. In 1764 perhaps the most notable account on punch-making was published in French: *Manuel Typographique* by Pierre-Simon Fournier^11^, which includes one of the most detailed descriptions of 18^th^-century punch-making available. Fornier was a remarkable French punch-cutter and typefounder. The next important references to punch-making are in the *Encyclopédie* by Diderot and d’Alembert (1752, 1763), which contains some explanations^12^ and plates illustrating this process^13^, although less detailed than those in the *Descriptions des Arts et Métiers*. Finally, it is important to mention both Johann Samuel Halle’s 1762 *Werkstäte der Heutigen Kunste, oder die Neue Kunsthistorie* (*Workshops of the contemporary arts, or the new art history*)^14^ and volume 6 of the *Dizionario delle Arti de’ Mestieri*, edited by Fassadoni in 1769^15^. Halle was a Prussian historian and engraver. These are perhaps the last important 18^th^-century descriptions of this craft.

Although all of these texts were contemporaneous to Baskerville, there are no records that might suggest that he was familiar with German or Italian, rendering the key and most accessible English and French texts the most likely sources that he might—or might not—have consulted before embarking into punch-making. In fact, in the 1750 June issue of *The Universal Magazine* there was an article on ‘The Art of Cutting, Casting, and Preparing of Letter for Printing’ that also included a plate representing a workshop. This article was a Moxon’s extract^16^. Whatever the case, these sources give us the general available background on punch-making at the time.

The explanations available in these volumes—including some written by printers and typefounders themselves—necessarily condense a very complex process into a few pages, which likely made it necessary to leave out details and deviations from the general norm. Furthermore, punch-making can be considered a trade secret perhaps even hidden from other workers in the printing house. It is therefore unlikely that anyone would publish a complete guide giving away all the specialised knowledge. Considering this, different written sources might complement themselves in this regard, providing different pieces of information on the process. Studying the Baskerville punches to reverse engineer the process of punch-making according to the tool marks left on them also offers complementary and novel nuance on the topic.

To do so, it is finally necessary to introduce the concept of *chaîne opératoire*, which has been broadly used by archaeologists interested in ancient technologies as a key tool to frame their research on material culture. A *chaîne opératoire* is a detailed account of the sequence of specific actions necessary to manufacture an object or perform a specific technological process. Defining them is a crucial step, because it allows us to further investigate recipes of production. Recipes are distinct units of cultural transmission that unite the raw materials, tools and installations necessary to manufacture an object, the *chaîne opératoire*, and crucially, the rules used to make decisions along the process and solve problems that might arise^17,18^. Understanding the latter is fundamental to contextually explain technological choices, as related to environmental, socio-economic and/or performance factors^19^. When recipes are consolidated, these conform technological traditions: fixed ways of doing things that extend over time, and sometimes, across space^20^. Thus, technological traditions can involve a single family, group of artisans, workshop or workshops and extend a decade or even millennia.

In this study, we reverse engineer the *chaîne opératoire* of punch-making and identify the recipes of production of the Baskerville punches. This ultimately allows us to define the technological tradition followed at the Birmingham workshop and better understand the logic behind the choices made at different stages of production. This is a level of depth currently unavailable through the study of contemporaneous texts on punch-making, one that can only be achieved through the application of heritage science techniques in combination with practice-based research and historical sources. This will ultimately serve not only to better understand 18^th^-century practices of punch-making, but also provide a unique perspective into the logistics and ways of doing of the Birmingham workshop that can be deployed to other collections of typographic punches.

## Methods

### The analytical study of the Baskerville punches

The current collection of Baskerville punches hosted at the Cambridge University Library has 3,240 items (Fig. 1). A detailed catalogue is being completed, as is an extensive photographic record of the punches. For this study, we measured the length of 2582, and the height and width of 599 punches. These measurements served to give a general idea about the shape of the metal bars that arrived in the Baskerville workshop. After that, 64 punches (see Supplementary Table 1 in Supplementary Materials 1) were selected to cover macroscopic variability as well as features of typographic interest (e.g. the characteristic Baskerville Q) and taken to the Archaeological Science Laboratories at the University of Cambridge for further analyses.

These 64 punches were typologically classified based on their macroscopic features and tool marks on the body of the punch to better understand the forging process involved in shaping the blanks and the initial stages of hammer end shaping. Next, their faces were observed both under stereo (Leica M205C) and digital microscopes (Keyence VHX-6000) under plain (PPL) and cross-polarised light (XPL) to record the tool or ‘witness marks’ related to letter-cutting and face-polishing, as well as identifying wear and tear. A subset of 12 punches was µCT (micro-computed tomography) scanned (SkyScan1273, 110 kV source voltage, 300 µA source current, Cu 0.3 mm filter, 15 µm pixel size) by Giuseppe Castelli at the same laboratories and radiographed by Susanna Pancaldo at the Fitzwilliam Museum (3 mA, 100 kV, 20 s). Both techniques helped investigate the presence and cause of internal cracks, the possible use of counterpunches during letter-cutting, and the absence of metal welding. The µCT data was visualised using Dragonfly software. Furthermore, FTIR (Fourier transform infrared spectroscopy) was conducted with the assistence of Catherine Kneale on another sub-set of three punches to characterise the coating substance present on many of them (iS5 Nanolet, iD7 ATR-Diamond, 16 scans resolution). Finally, to inform this research, we used experimental replications of the process of cutting blanks and forging performed by Annie Higgins at the School of Jewellery, Birmingham City University. A practical metal engraving workshop led by Anastasia Young, also at the School of Jewellery of Birmingham City University served to draw parallels of engraving witness marks between newly-made and the historical artefacts.

With the complementary information provided by these techniques and practical experiences, it was possible to reverse-engineer key stages of the *chaîne opératoire* of punch-making. These were then discussed in relation to 17^th^- and 18^th^-century accounts of punch-making. We focused on the English and French printed texts^6,8,11,12^.

We use the term ‘Baskerville punch’ to refer to any punch that has a letter cut with this typeface. The term ‘Birmingham punch’ is reserved for those punches produced at the Baskerville workshop in Birmingham during the 18^th^ century. ‘Modern additions’ refers to those punches clearly manufactured during the 19^th^ and 20^th^ centuries, bearing a series of obvious macroscopic characteristics that easily set them apart, as described in the following section. There are, however, some punches that clearly display some, but not all, of the typical Birmingham characteristics while at the same time they do not look like the 19^th^- and 20^th^-century additions. Those, likely manufactured soon after Baskerville died, are referred to as ‘early historical additions’. The Birmingham punches together with these early historical additions constitute what we call the ‘core of the collection’.

## Results

In this section, we describe the operations of punch-making that we can infer based on the examination of the objects. Later in the discussion, other stages of manufacture not so visible on the finished objects but known from historical sources or current accounts are also integrated into the *chaîne opératoire*.

### Blanks acquisition and shaping

The process likely started with ready-made, long, square-section iron bars that would have arrived in the workshop from different procurers, as suggested by the distinct manufacturer stamps preserved on the surface of some of the largest punches. These stamps are currently under study and will be the subject of a future publication together with further compositional and microstructural data that will allow us to approach 18^th^-century iron and steel manufacturing techniques.

**Fig. 2:**
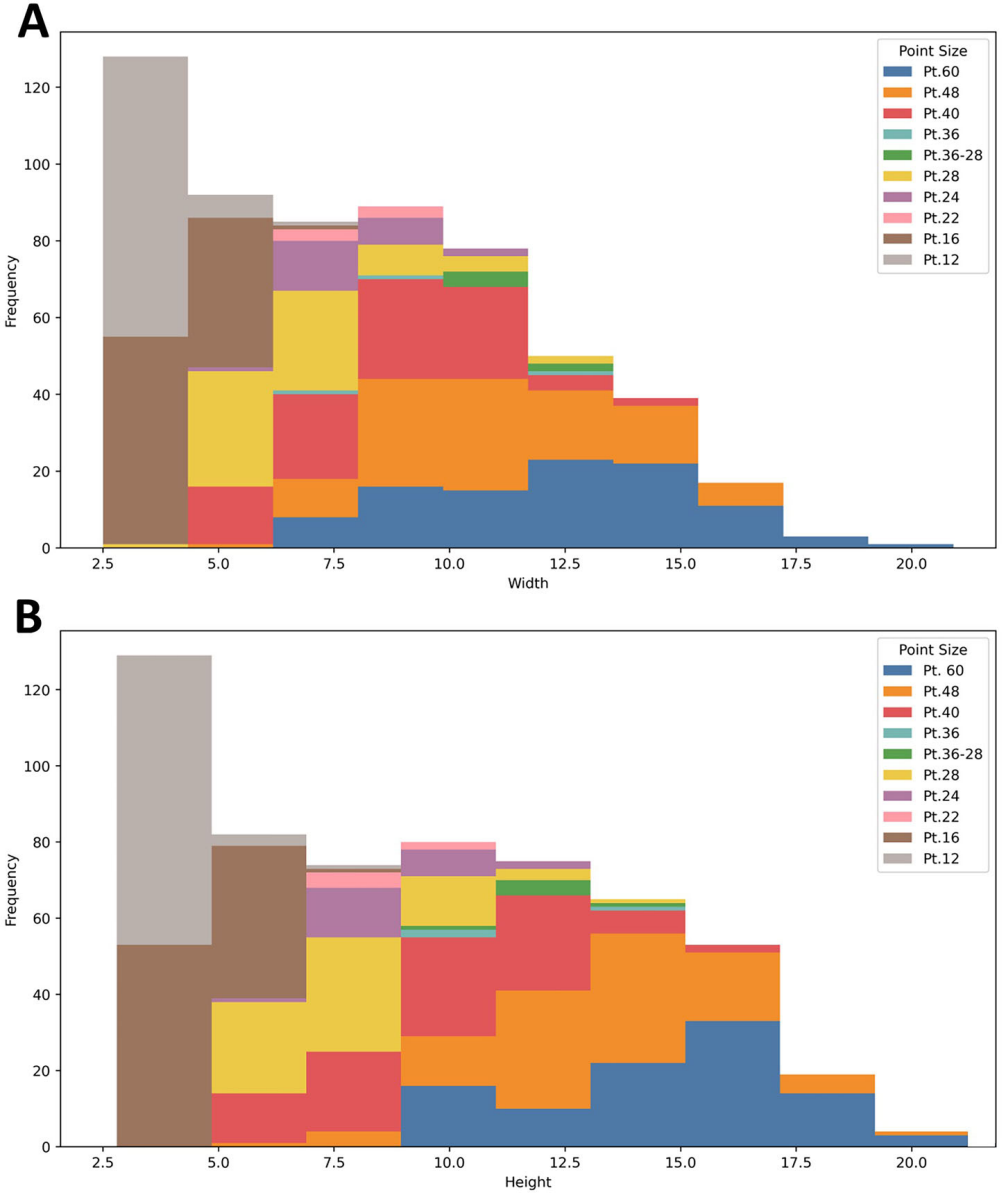
Stacked histograms showing the width and height (mm) of punches of different point sizes. **A** Width. **B** Height.

Although the iron bars were then cut and hammered to modify their shape (see below), the height and width of the punches close to the hammer end are expected to be close enough to those of the original bar as this is the area that required less reshaping. Thus, when plotting the measurements of these sections of the bars for individual point sizes we can generally see that larger point sizes required larger bars (Fig. 2). This likely entailed receiving metal bars of generally standardised dimensions that were worked in batches to produce the necessary blanks. Interestingly, the data shows that a same bar size could suit multiple point sizes, as the original dimensions were adjusted during the forging stage (see below).

**Fig. 3:**
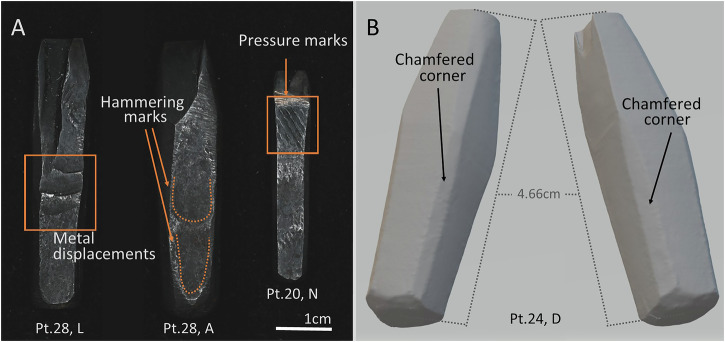
Photographs and µCT 3D model of selected punches showing different tool marks related to forging. **A** Examples of metal displacements, hammering marks, and pressure marks. **B** Example of chamfered corners.

The process continued at the forge, as evidenced by the witness marks visible on the body of the punches, especially the largest ones. The commonest marks are metal displacements, and hammering and pressure marks left by clamps and vices used to hold the blanks (Fig. 3A). These tool marks result in uneven surfaces and are visible across the body of the punches except close to the face, where filing marks related to letter-cutting have removed them.

The overall shape of the punches allows us to easily identify three different ways in which the craftspeople prepared the surface where the character would be cut.

**Fig. 4:**
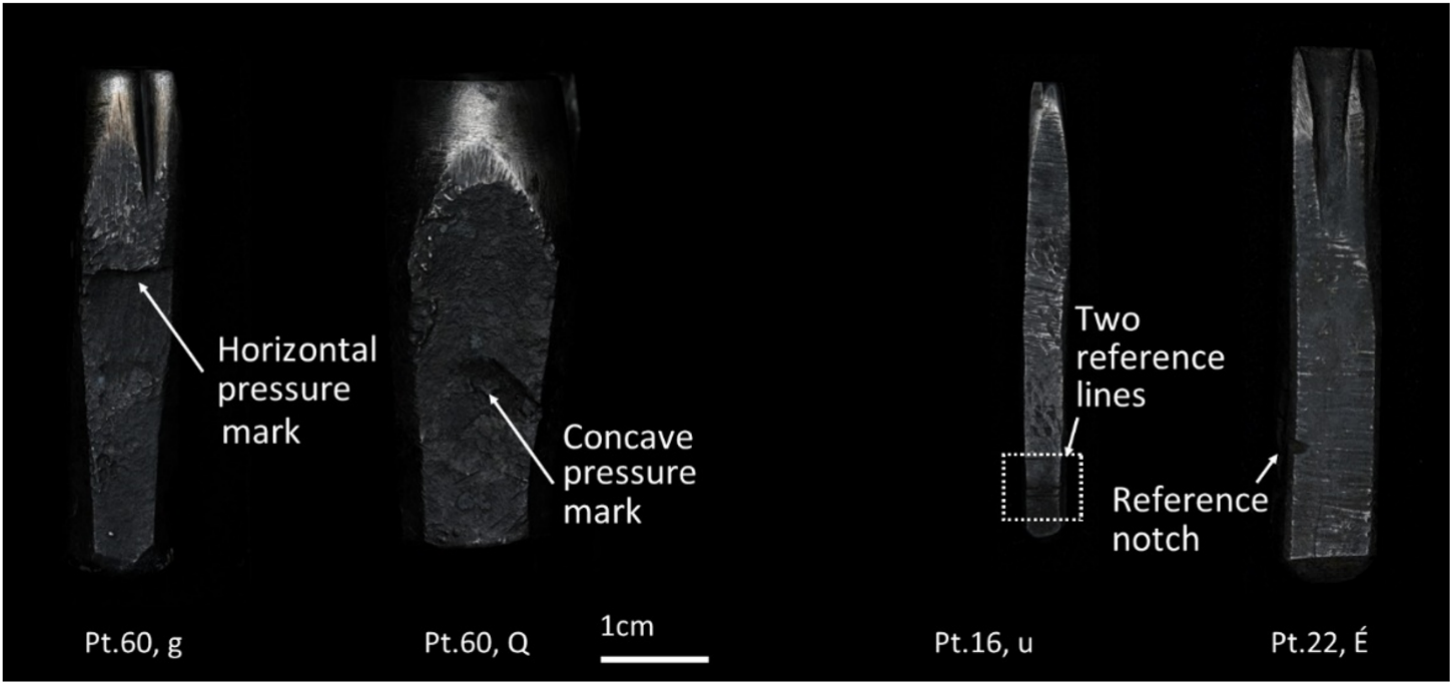
Photographs of selected punches exemplifying tool marks related to the upsetting process and different reference marks for different purposes.

**Fig. 5:**
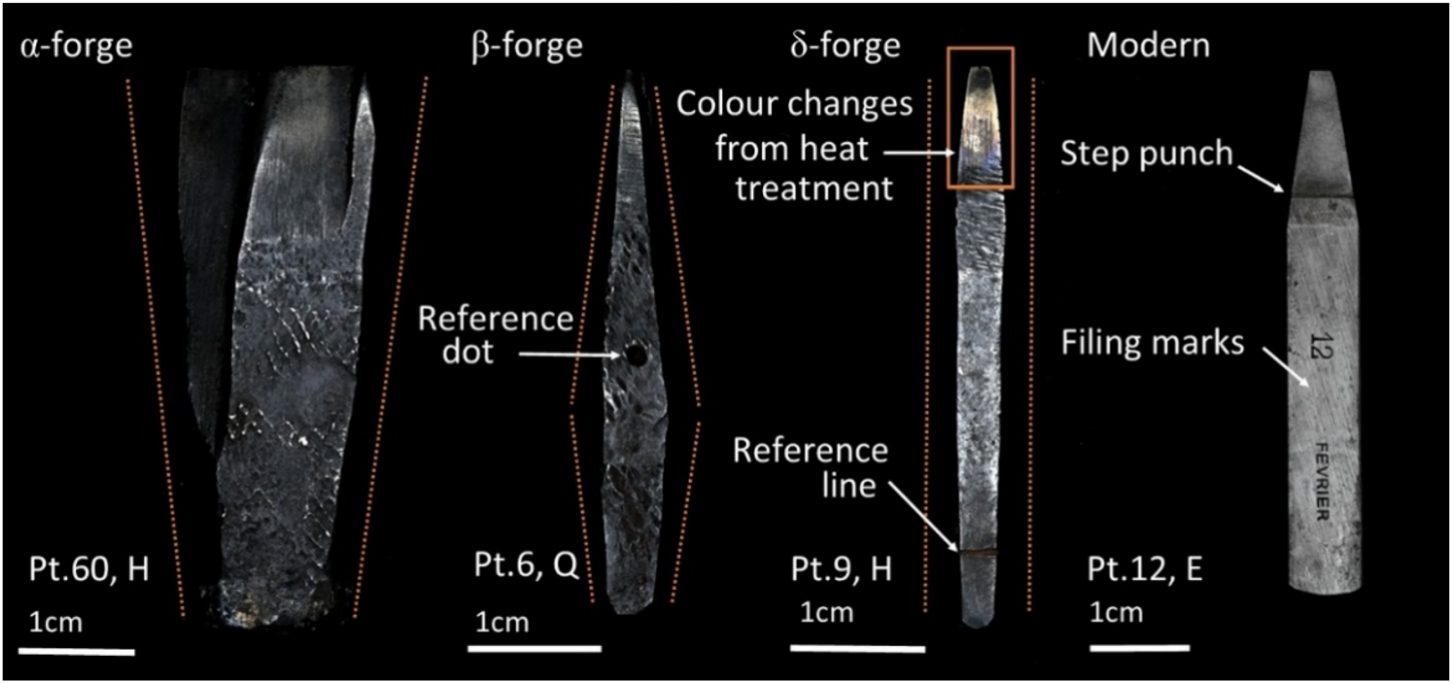
Examples of punces forged following the α, β and δ pathways, and a modern addition with no evidence of forging.

**Fig. 6:**
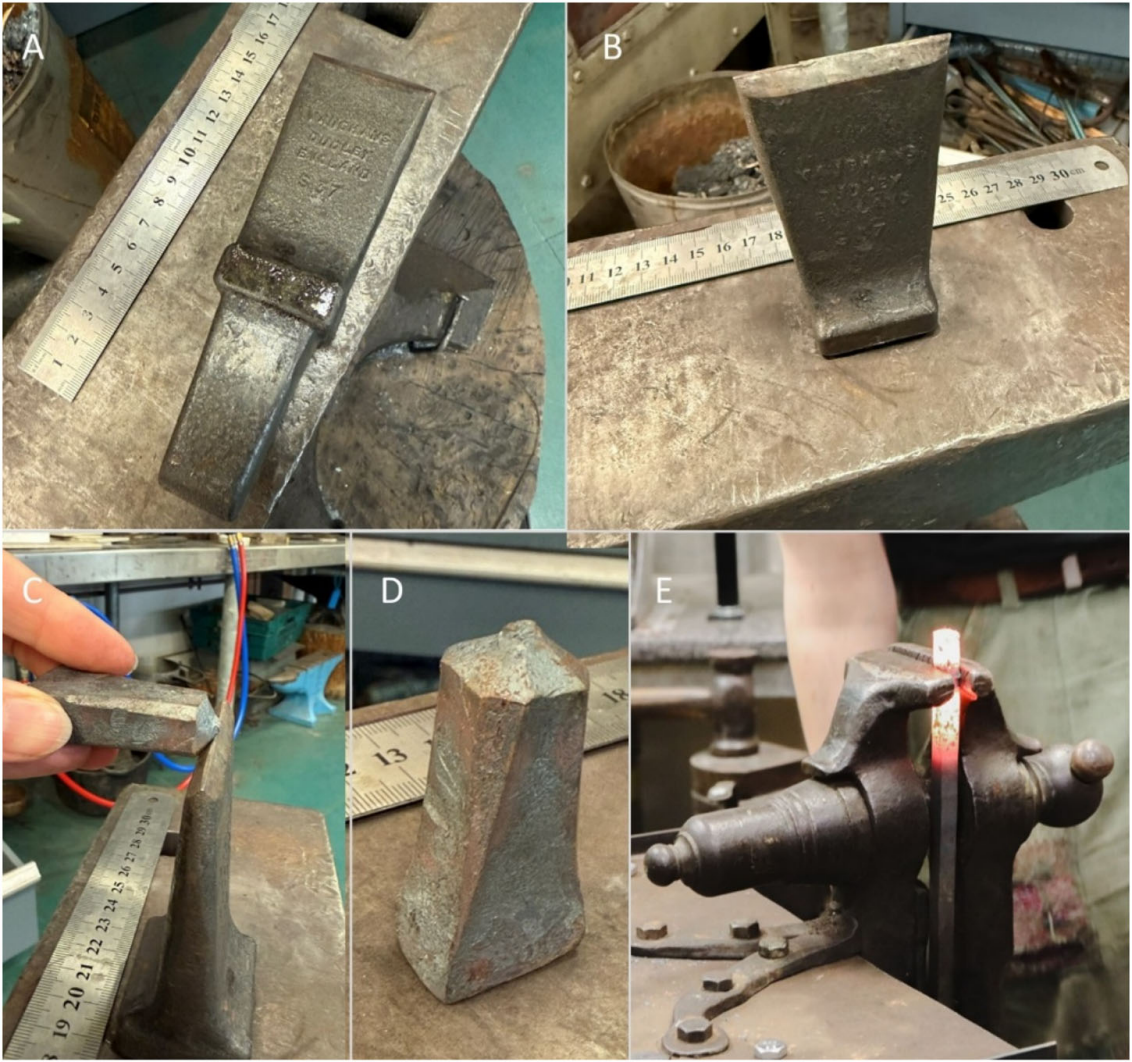
Different stages of reproductions at the forge. **A** Hot cut hardy on an anvil. **B** Hot cut hardy installed in the hardy hole of the anvil. **C** An already cut piece of metal next to the sharp edge where it was struck until cut. **D** Resulting shape at the hammer end of the blank after cutting it using the hot cut hardy. **E** Setup for metal upsetting.

In some cases, the letter to be cut was considerably larger than the surface naturally provided by the metal bar. When this happened, the blacksmith followed what we call here the α forging pathway. Starting with the metal bar, the hammer end was slightly forged to a taper. Then, the rod was cut at the necessary length and the area where the future face of the punch would be located underwent a process called ‘upsetting’. This involves hammering the top surface of the punch (the future face) while it is held vertically to displace metal and create a larger surface that fits a bigger design. While this is done, the punch would be held by a blacksmithing leg vice, which would compress its lower body (Fig. 6E). Depending on the type of holding, it is sometimes possible to observe remaining tool marks related to this process: horizontal and concave pressure marks when the blanks were held by their sides, and pressure notches when they were held at their corners (Fig. 4). As upsetting implied alternating between hammering the sides of the punch and the face several times, in some cases these tool marks have been considerably smoothed out. Regardless, upsetting resulted in punches with a characteristic torch-like section that have a thicker face than hammer end (Fig. 5). This thickened area likely also contributed to their durability, as it was better able to absorb the impact of repetitive strikes during subsequent punch use. This α forging pathway is generally restricted to the largest point sizes, from 60 pt down to 12 pt (only one case in 12 pt), consistent with the need to create larger working surfaces. Examples of α-forge punches are listed in Supplementary Table 1 in Supplementary Materials 1.

Upsetting punches is briefly described in the *Manuel Typographique*^11^, and it is especially mentioned for cases in which the metal bar was not big-enough to fit the character: ‘*Lorsqu’on ne trouve pas d’acier en barre assez gros pour former ces lettres, on le fait refouler par le bout, & le reste de la tige deviant plus menu, ce qui n’en vaut mieux*’. Fournier also proposes an alternative way of executing these big punches by welding a piece of iron to a piece of steel to then again upset the metal so the area where the character will be cut (the steel) is larger than the iron hammer end: ‘*ou bien l’on fait souder de l’acier sur des bouts de fer. […] ensuite on coupe le fer de la longueur que doit avoir le poinçon, & on forge la tige de façon qu’elle soit plus petite par un bout que du côté de la lettre*’. The radiographs conducted on a selected number of punches, including some α-forge ones, (see Supplementary Fig. 9 in Supplementary Materials 1), show no welding lines, so the Birmingham workshop does not seem to have relied on this approach.

Other punches, however, followed the β forging pathway. This forging pathway was used when the surface of the original metal bar was slightly smaller than the letter to be cut. There are two ways of proceeding to create the characteristic canoe-like shape of these punches (Fig. 5). The first one is somewhat more complicated than the latter, and involves starting with the α forging pathway followed by the thinning down of the upper part—where the face was to be cut—to the required size after upsetting the blank. This however would have likely involved constant bending that needed to be corrected. Alternatively, forged tapers can be drawn from a thick rod towards both ends, leaving a bulged section at the central part of the blank. In many of these punches a circular mark was made on one of their sides, always close to the thickest part of the punch (i.e. the shoulder) (Fig. 5). These are likely reference marks that indicated to the blacksmith the area from which they needed to start thinning the metal bar. These marks have not been found in punches following different forging pathways. The thicker middle area of β-forge punches likely helped prevent bending and cracking derived from continuous use, while at the same time it likely facilitated handling during the striking process. β-forge punches span a wide range of point sizes, from 40 pt down to 8 pt. Examples of β-forge punches are listed in Supplementary Table 1 in Supplementary Materials 1.

Finally, other Birmingham punches were shaped following the δ forging pathway, reserved for punches that required minimal or no forging, as the general dimensions of the iron bar were appropriate to broadly fit the desired letter. δ-forge punches have, therefore, straight sections, with very minimal or no thickening of the bar (Fig. 5). Sometimes, the face of these punches retains the prismatic shape of the original bar, but other times it was reduced during punch-cutting to fit a slightly smaller point size. δ-forge punches cover the medium to small point sizes (16 pt to 6 pt). Examples of δ-forge punches are listed in Supplementary Table 1 in Supplementary Materials 1.

Crucially, during the forging processes, the flat surfaces of the four long sides were maintained, but the corners were chamfered. This process involved flattening the sharp 90° corners of the original metal bar to create a bevelled edge, and likely took place towards the end of the forging stage. Chamfering enhances the toughness of the bar and prevents cracks (Fig. 3B). Chamfered corners have been observed in all point sizes but not in modern additions. This therefore is a characteristic of the Birmingham punches and more generally, of early punch-making that, contrary to later 19^th^- and 20^th^-century examples, required forging (see the Discussion section).

Whatever the forging pathway selected, the long bars had to be cut in segments along the way (i.e. before upsetting in the α forging pathway, before drawing the upper taper in the β forging pathway, after determining the necessary punch length in the δ forging pathway). To do so, a hot cut hardy was likely installed on the square hardy hole of the anvil (Fig. 6A, B). The hot metal bar was then cut by placing it on the sharp edge of the hardy cutter and striking it with a hammer. The bar was rotated in between strikes to facilitate a clean cut (Fig. 6C). This resulted in pyramid-like protuberances at both ends of the separated sections. While for the detached blanks this protuberance was accordingly worked as described in the forging pathways above, in the remaining long rod, this pyramid-like protuberance would correspond to the hammer end of the next punch blank to be extracted from the rod (Fig. 6C, D). We will later see how this pyramid-like protuberance at the hammer end was filed down in later stages of punch-making (see below).

**Fig. 7:**
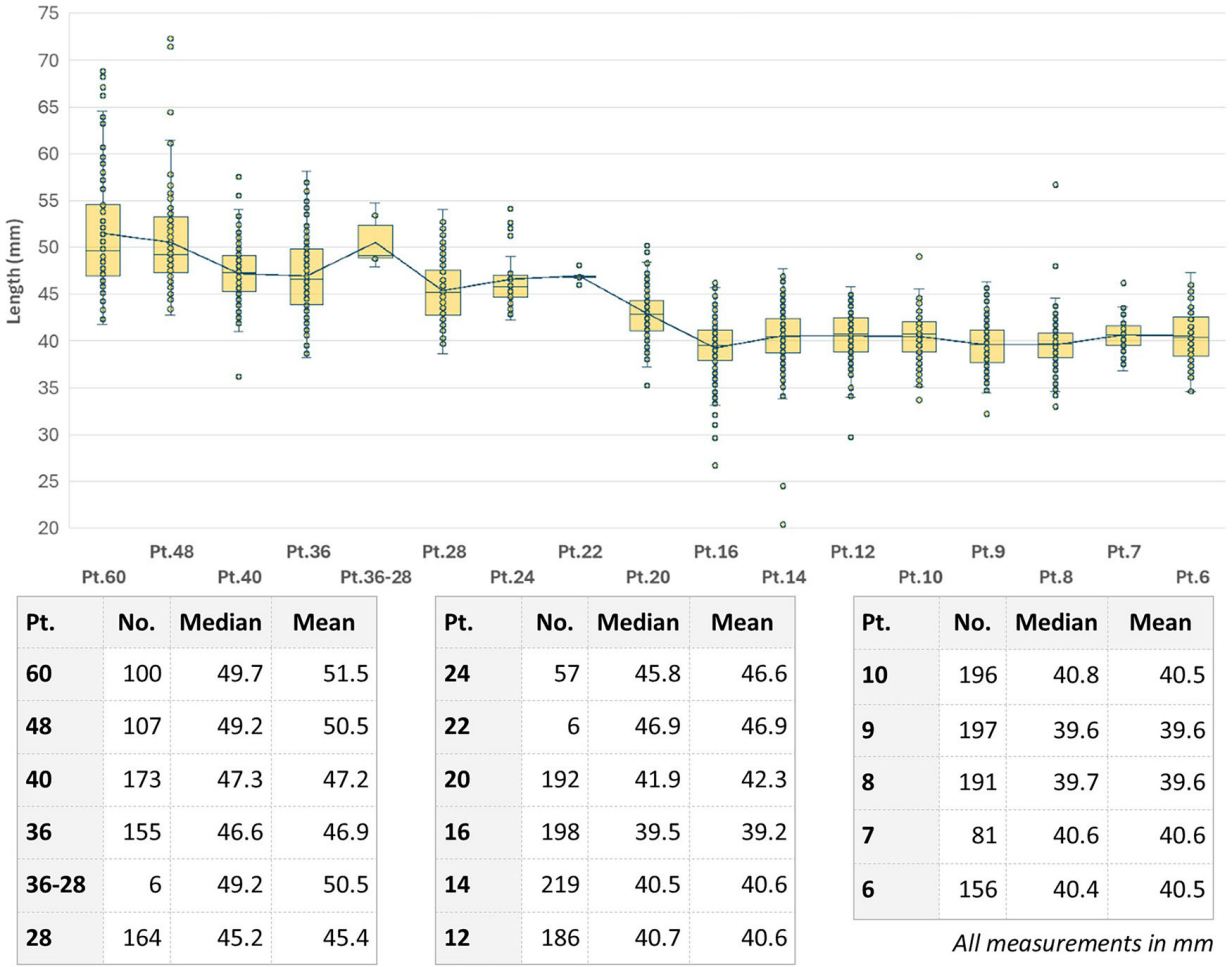
Length of the punches studied, and median and mean length per point size excluding obviously modern additions. The continuous line represents the mean.

When plotting the lengths of all the punches measured, excluding the obviously modern additions (*n* = 2384), it is possible to see that the bars employed for 60 pt, 48 pt and an uncharacterised point size between 36 pt and 28 pt were cut in ~5 cm segments, whereas 40 pt, 36 pt, 28 pt, 24 pt and 22 pt are cut at ~4.5 cm, and 20 pt and below are cut at ~4 cm (Fig. 7). This indicates the use of consistent cutting criteria depending on the point size and the likely use of guiding tools to measure these dimensions consistently. Despite this, some outliers can be identified, like the 16 pt roman Q that is only 3.5 cm in length. When observed closely, its hammer end shows evidence of reworking, probably due to heavy use (see “Wear & Tear” section)—a process that likely shortened its original length. Another exception is 60 pt roman 6, measuring 5.8 cm. This punch, when scrutinised, was identified as a potential early historical addition given some specific macroscopic traits that will be discussed in the following sections (i.e. flat hammer end not filed, extensive use of engraving in a big point size). Thus, striking differences in length among punches within the same point size can be indicative of a later addition—during or after Baskerville times—or reworking episodes.

To conclude this section, it is necessary to briefly compare the Birmingham punches to the modern additions. The latter show much more regular, uniformly parallel surfaces, consistent with the proposition that the bars were machine-made by rolling. As they were not forged manually, their lateral edges remain in a 90° angle. These machine-made bars only needed to be cut and roughly filed across all the surfaces, creating a smoother finish (Fig. 5). In the same vein, unlike β- and δ-forge punches, the shoulders of the modern punches were entirely created during the cutting of characters rather than by forging.

### Hammer end shaping and design transfer

**Fig. 8:**
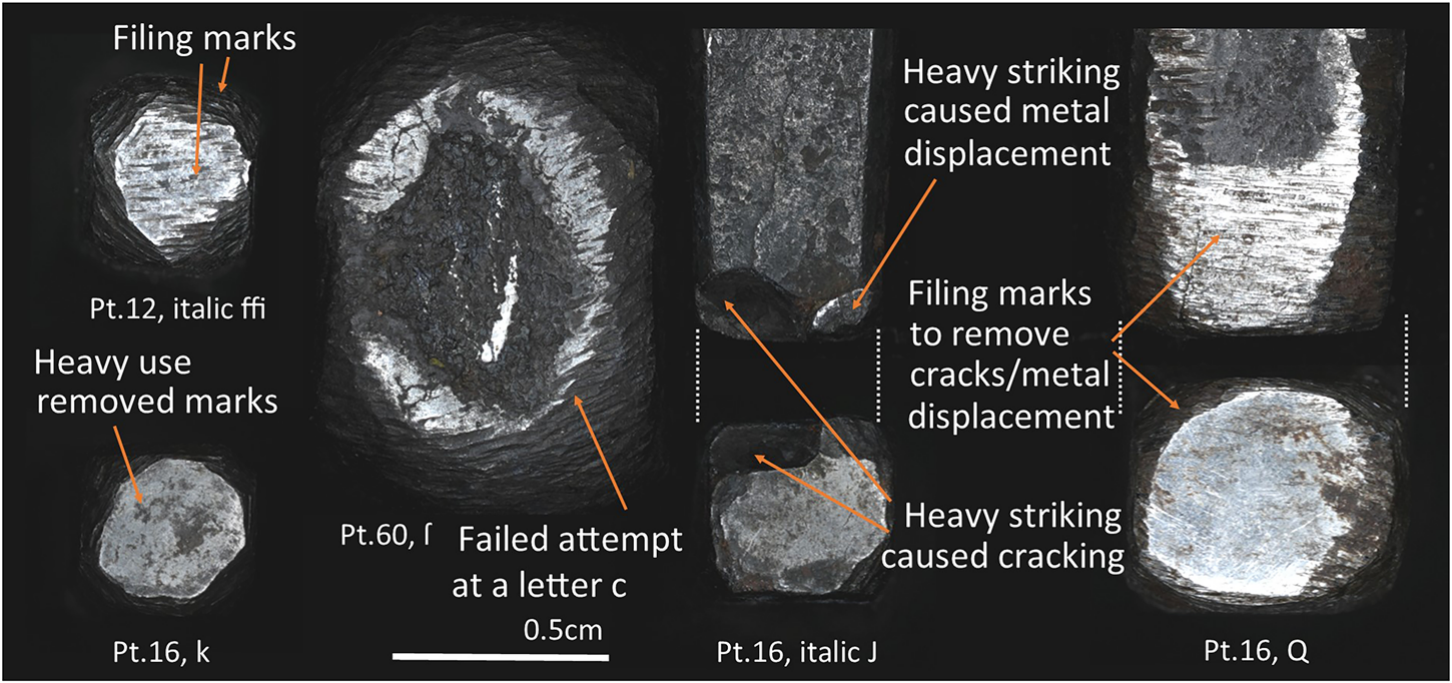
Photographs of the dome-shaped hammer ends of selected Birmingham punches with different tool marks and degrees of use and repair.

The next step for which we have evidence is hammer end shaping. At this point, the blank was held in some kind of clamp or support facing up or down depending on the area to be worked. The hammer end was likely the first part to be shaped, as the reverse order would risk damaging a finished cut letter. The pointed metal end resulting from the bar-cutting process (Fig. 3D) was rounded down with a coarse file, creating a dome shape with a flat top that would later be suitable for striking. On most of the Birmingham punches these filing marks are visible at the sides of the dome. However, the flat area of the hammer ends is mostly devoid of filing marks, as they were removed through use (Fig. 8). Generally, the shape of the dome-like hammer ends varies considerably: different facets are visible, different degrees of curvature, etc. This indicates that hammer end shaping was an expedient process.

The next visible evidence of production are reference marks across the bodies of some of the punches. For most letters whose design appears similar regardless of their vertical orientation, such as capital roman O’s, H’s, or I’s, the side of the punch which corresponded to the lower part of the letter was marked with a transversal guiding line referred to as ‘signature’ (Fig. 7)^21^. In some cases, two lines instead of one were cut (Fig. 6). Sometimes these signatures are not located at the side where the lower part of the letter was finally located, raising the question of whether they indeed always served that purpose, or whether they were sometimes ignored. In addition, some punches have small notches at one or several corners (Fig. 6), but are not associated to punches with signatures. Most of the punches with notches have some, but not all, of the diagnostic features of the Birmingham punches. It is therefore possible that they might be early historical additions cut close to Baskerville’s time but by a different hand. All these marks likely played a reference role during punch-cutting, as the *Encyclopédie*^12^ suggests, but they do not seem to follow a consistent pattern across the collection to infer their meaning.

**Fig. 9:**
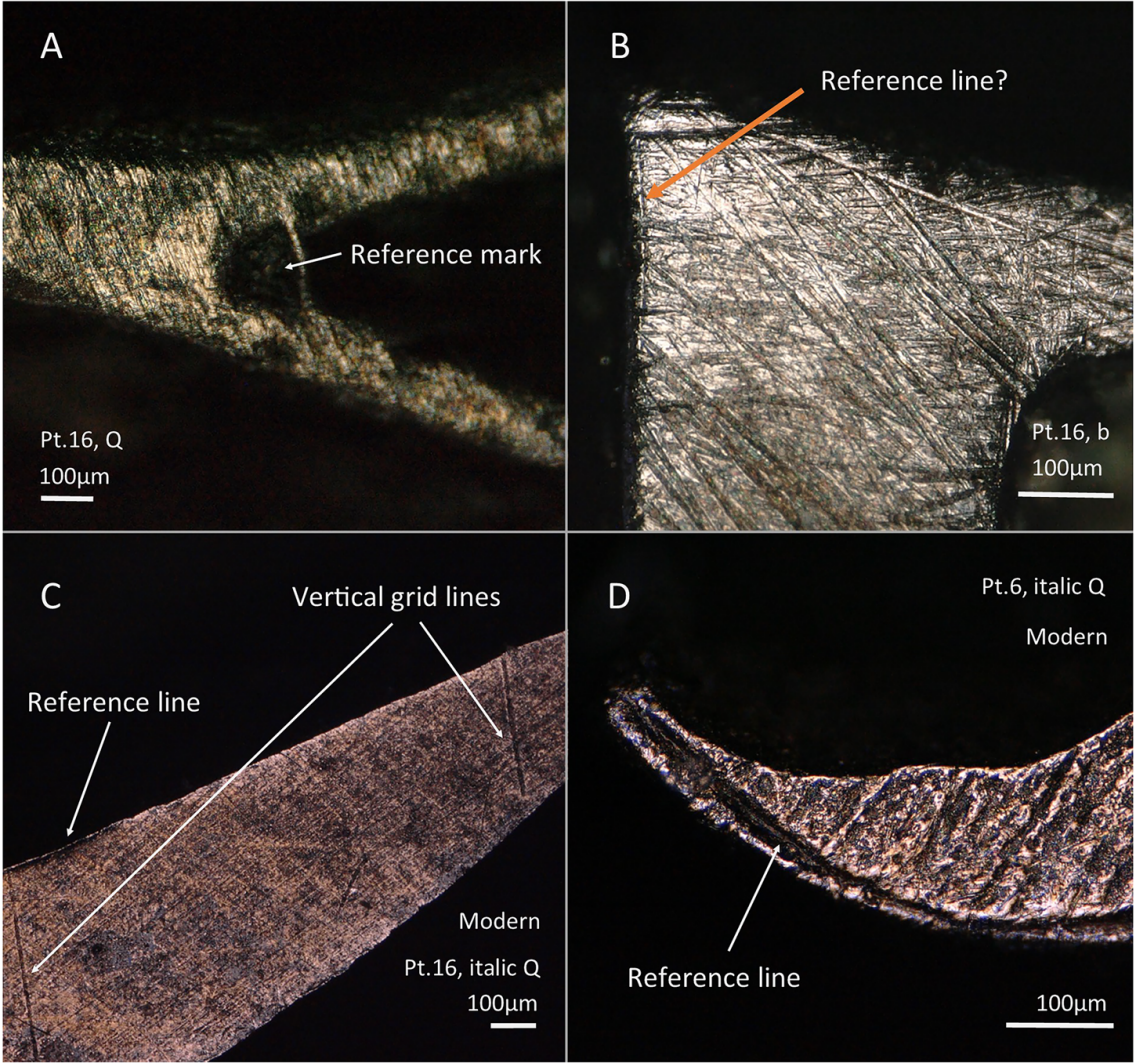
Evidence of design transfers on the Baskerville punches. **A**, **B** XPL micrographs of Birmingham punches with some potential reference marks for design transfer. **C**, **D** XPL micrographs of modern punches with clear reference marks for design transfer. The punch in C was cut by Plumet as his surname is written on it.

At this point it is likely that preparation of the face of the punch started, making it flat with a file and polished, but no traces of this stage remain in the Baskerville punches as they were obliterated during subsequent letter cutting. In addition, practically no evidence corresponding to the process of transferring the design onto the punch has survived among the punches studied under the digital microscope (*n* = 64). Only two possible exceptions were identified, where guiding marks are visible. The first is 16 pt roman Q, where at the internal angle at the upper part of the tail of the letter, a mark was made, presumably as a reference point, for the location of this angle (Fig. 9A). The second is 16 pt roman b, where the ascending stroke seems to follow a straight reference line (Fig. 9B). Notwithstanding these exceptions, the general lack of reference lines is a peculiarity of the Birmingham punches, contrasting with some of the modern additions identified in the collection that bear clear reference lines, including grids (which are consistent with Koch’s description of the punch-making process in the 20^th^ century^21^) and lines following the shape of the letter (Fig. 9C, D). This suggests that different strategies were used at different times and/or that the Baskerville workshop removed such marks.

### Letter-cutting

Letter-cutting was largely achieved through a combination of engraving and filing. However, before presenting a detailed description of this, it is necessary to start by evaluating the possible use of counterpunches as the initial step in the making of characters. These punches were exclusively used to create the ‘counter’, i.e. the enclosed or partially enclosed negative space of a letterform in a punch, such as the inner spaces of O’s, Q’s or e’s, as well as the lower inner areas of e’s or H’s, which are partly open. These areas are the first parts to be cut, regardless of whether counterpunches were used.

As grease and dirt prevented direct observations of these areas with a microscope, µCT scans were obtained from a selected number of punches (*n* = 12). The short µCT videos (see Supplementary Movies 1 to 12 and Supplementary Note 1 in Supplementary Materials 2) show how these inner spaces have uneven bottom surfaces and open progressively, which *a priori* seems more consistent with the use of engraving rather than counterpunches. However, the *Manuel Typographique*^11^ offers an interesting perspective that might help interpret these observations: while counterpunches are recommended for standard point sizes (i.e. around 16 pt), the *Manuel* recognises that the largest point sizes raise challenges because of the greater resistance of the metal and the larger metal displacements, requiring multiple filing and polishing stages. In these cases, two alternative pathways are recommended.

The first involves using a counterpunch to very lightly leave a guide imprint on the face of the punch; then, a twist-drill is used to start perforating the inner side, followed by the use of different gravers to cut the inner angles of the letters; the counterpunch is used again once the general shape has been carved, to make the perfect imprint. The second solution is to completely avoid counterpunches and use the twist-drill and gravers for the whole process. This last option is favoured both for the largest point sizes (which agrees with Moxon^6^) and the smallest ones, as it is unpractical to make suitable counterpunches for the latter. In 20^th^-century accounts of punch-making, drilling, counterpunching and engraving are also mentioned as methods to make the inner space of the letters, sometimes used in combination^21^.

In the Birmingham punches studied, the uneven surface of the inner spaces, sometimes with a deeper area in a corner, seems consistent with the use of a twist-drill to start opening those spaces before using gravers. Despite this observation, we cannot conclusively rule out the use of counterpunches, as they might have been used in combination as described by Fournier. No counterpunches have been identified in the whole Baskerville collection. Whatever the case, it is very likely that, after shaping the counters, refiling/polishing of the face flat was necessary before proceeding to cut the outer contours of the letters.

To cut the outer part of the letters, it is likely that metal was initially roughly filed, cut, or scooped out with chisels in the areas of the blanks where more material needed to be removed. However, not much evidence remains of these initial processes, as most of the visible tool marks correspond to the last steps that focused on the careful shaping of the letters. The selection of punches analysed indicates that this process mainly involved two different techniques for the outer contours: filing and engraving. Filing is the manual removal of small amounts of metal using a file, i.e. a hardened steel tool with parallel ridges or teeth. Files can be of varied shapes (e.g. flat files, needle files, round files, etc.) and coarseness so it is possible to shape the metal with the necessary precision. Engraving, on the other hand, is the process of cutting or incising a design into the metal using a sharp tool called a graver. The working end of gravers can also vary in shape.

**Fig. 10:**
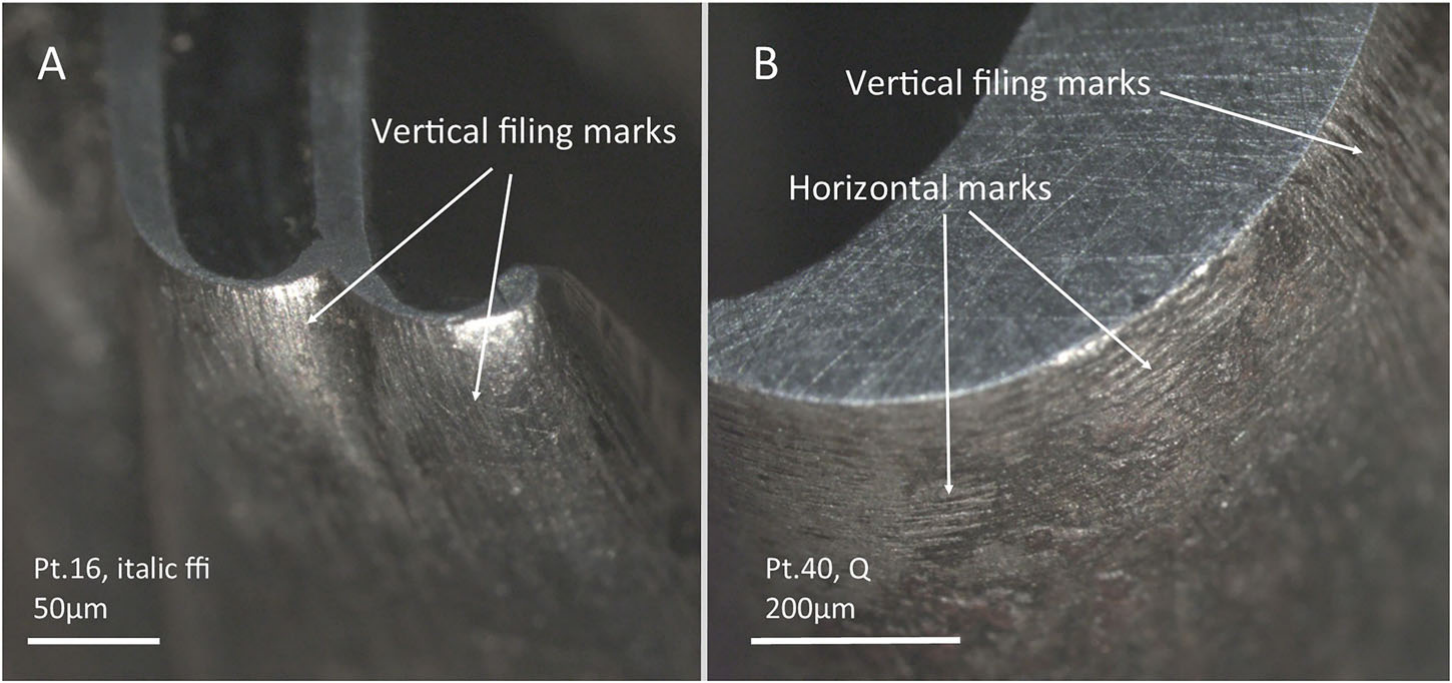
Stereo-micrographs of selected punches with evidence of filing. **A** Vertical filing marks. **B** Combination of vertical and horizontal filing marks.

Filing was used to cut the outer part of the letters, likely using coarser files at the beginning of the work and finer ones as they approached the desired outline. Filing was mostly conducted vertically, moving the tool up and down parallel to the long axis of the punch. However, examples of horizontal filing, that would require the tool to move parallel to the punch, have been also observed, mostly related to the external curvatures of letters such as O’s or Q’s. Vertical and horizontal filing was sometimes combined. Both types of filing strategies produce distinct parallel lines visible under the microscope (Fig. 10).

**Fig. 11:**
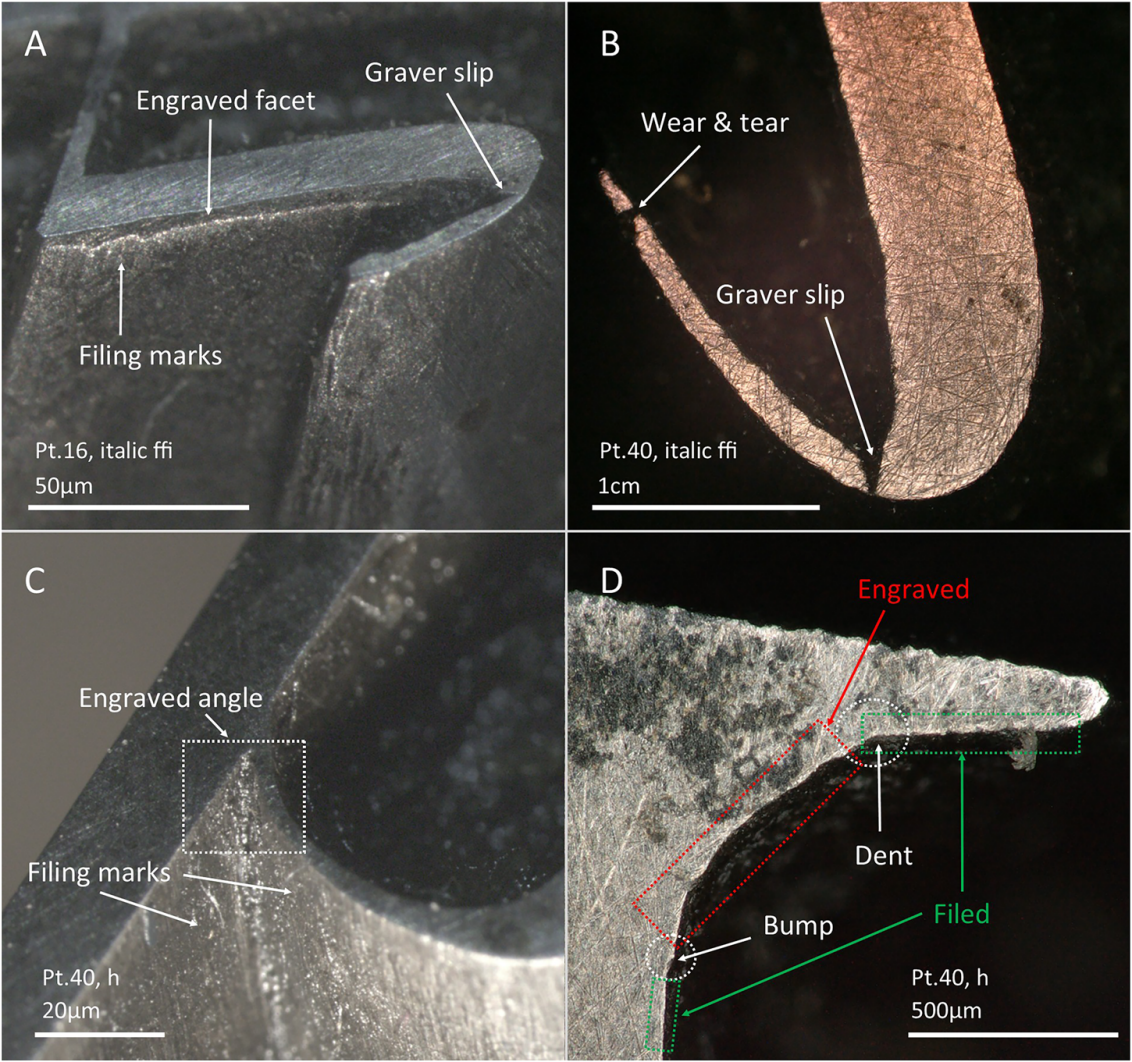
Stereo XPL and PPL micrographs with evidence of combined used of filing and engraving. **A** Engranved facet. **B** Evidence of graver slip. **C** Engraved angle surrounded by a filed area. **D** Combination of filing and engraving at an ascender inner angle.

Filing was generally used when the letter was big-enough and had relatively easy angles. When one of these prerequisites was not met, it was necessary to integrate engraving. However, it is very common to see the use of engraving in combination with filing. This entailed filing as close as possible to the desired external shape of the letter and then finishing the outer contour by engraving. This created a perpendicular facet of different dimensions depending on the working angle (Fig. 11A). We found no tool marks consistent with progressive strikes in these facets that might indicate chiselling, so they were most likely made using burins and sculpters.

**Fig. 12:**
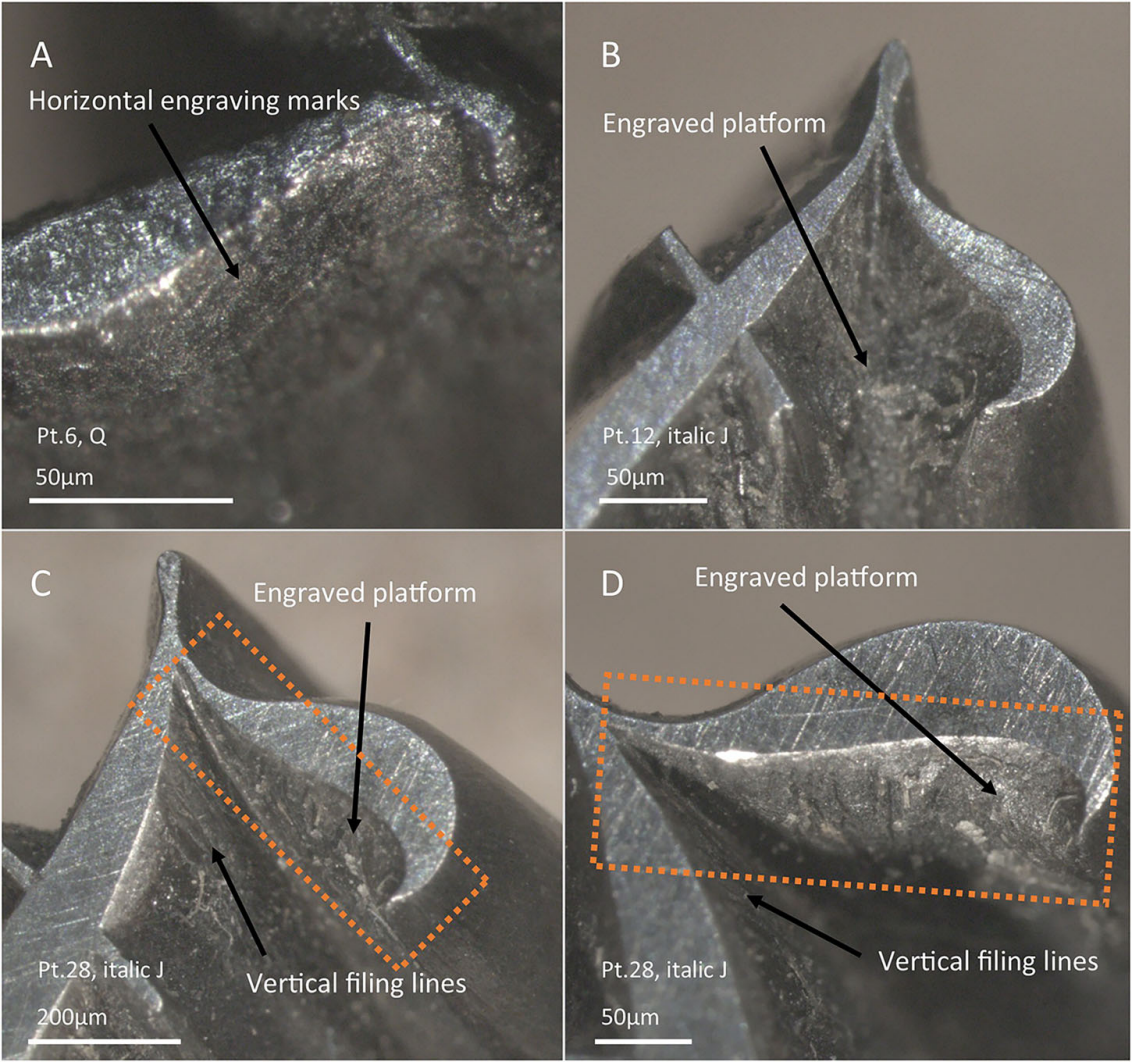
Stereo-micrographs of selected punches showing evidence of engraving. **A** Horizontal engraving marks. **B**–**D** Engraved platforms of different sizes. Note that in (**B**), the inner part of 12 pt italic J was cut with a large engraved platform, whereas for 28 pt italic J (**C**, **D**), this area of the punch was cut differently, with most of the area shaped by filing and only a small space (dotted square) shaped by engraving. The dotted area in **C,** **D** shows the same area of the same punch.

**Fig. 13:**
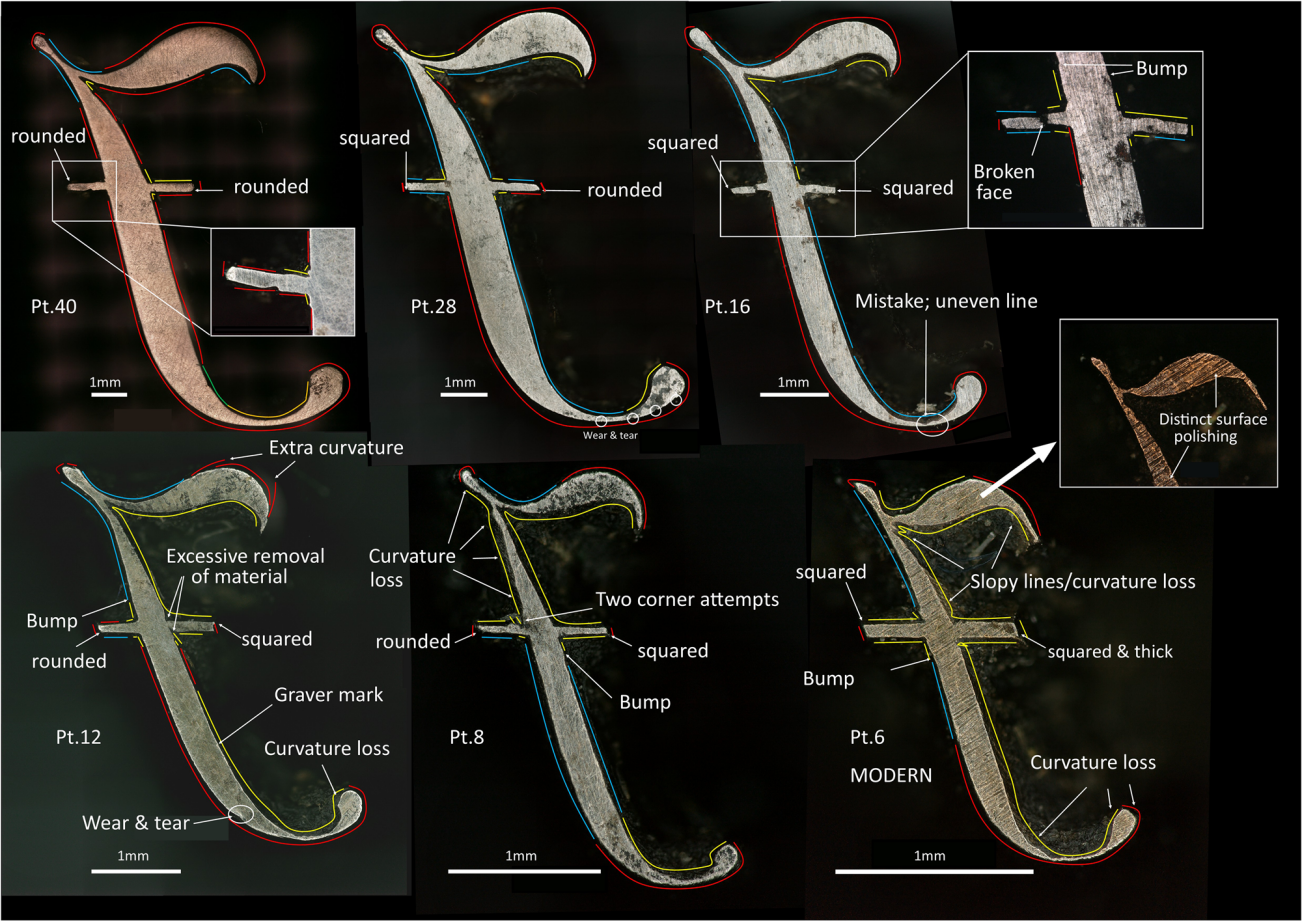
Comparison of the techniques used to cut italic J’s of different point sizes. Red lines indicate areas of the punch cut by filing, yellow lines indicate areas of the punch cut by engraving and blue lines indicate areas of the punch cut by a combination of filing and engraving.

Finally, when the letter to be cut (or a specific area of a larger letter) was too small to use a file at all, engraving was the only solution. This is usually the case for the acute corners of big point sizes (Fig. 11B, C), or for relatively larger parts of letters as the point size is reduced (see below). The sole reliance on engraving is denoted by distinct horizontal marks, usually less smooth than those related to filing (Fig. 12A). Sometimes, it is also possible to infer sole reliance on engraving when observing platforms a few millimetres below the face of the letter, indicating the depth to which the punch-cutter engraved the letter (Fig. 12B, C). It is important at this point to differentiate these platforms from those in internal areas of letters created by counterpunches, as described by Smeijers^22^. In the case of the Birmingham punches, the bottom part of these platforms is irregular, sometimes clearly showing metal scooping at the bottom (Fig. 12B, D) and/or graver marks or slips at the edges of those platforms (Fig. 12C). The edges between the bottom and sides are usually not well-defined, and the depth of these platforms is small (e.g. Fig. 12B has about 50 µm depth), which is also consistent with the use of gravers^22^. No counterpunch platforms of the type described by Smeijers have been observed in our sample. Interestingly, when relying on engraving, in those areas difficult to access, it is possible to still see small deviations of the tool in the shape of the letter (Fig. 11A, B). Moreover, where there is a change between engraving and filing techniques, it is common to see a bump/dent that indicates the change in tools (Fig. 11D and Fig. 13).

Having established the diagnostic witness marks for either filing or engraving, we recorded them systematically across a selected number of punches (*n* = 64). When observing the same letters in decreasing sizes, it is possible to see a progression in the use of these techniques. The largest point sizes are generally cut by filing and occasionally combine filing with engraving in some areas. As the size of the letter decreases, there is a stronger presence of the combination of filing and engraving (i.e. presence of engraving facets). Finally, the smallest point sizes were mostly cut using engraving. This pattern has been generally seen in all the characters for which this type of analysis has been conducted: i.e. italic J’s (Fig. 13), roman and italic Q’s, roman h’s, italic ffi’s, roman ?'s, roman 6’s and roman and italic g’s (see Supplementary Figs. 1 to 6 in Supplementary Materials 1). A good example of the adaptation of the technique to the shape and size of the letter is provided by the italic J’s (Fig. 13). While 40 pt, 28 pt and 16 pt had the inner top right part cut by filing or a combination of filing and engraving (with targeted engraving of the acute corners), from 12 pt and below, this area is too inaccessible, so it had to be completely done by engraving, leaving a distinct platform (Fig. 11B–D and Fig. 13). There are, however, exceptions to this progression from filing to engraving, indicating perhaps that decisions were made *ad hoc*. For example, the lower part of the tail of the 8 pt roman Q was engraved, whereas the 6 pt had this area executed by a combination of filing and engraving (see Supplementary Fig. 4 in Supplementary Materials 1).

When comparing the Birmingham punches to modern additions, we can see that, generally, the Birmingham punches tend to show that filing was used as much as possible, either on its own or in combination with engraving in the smallest point sizes. The sole use of engraving is strictly constrained to areas of the letter that would be impossible to access with a file. In the modern punches, conversely, we can see a higher preference for engraving—a practice at odds with some 20^th^-century accounts claiming that engraving the outer part is only used occasionally^21^. For example, in Fig. 13, the 6 pt, italic J (a modern punch) uses engraving in the inner part of the descender. Instead, the 8 pt of the same letter, which is a Birmingham punch, still combines filing with engraving in this area. Further examples can be seen in Supplementary Materials 1. Thus, this reliance on filing seems to be a characteristic of the technological tradition followed at the Birmingham workshop.

There are other technical aspects of the Birmingham punches that reinforce the impression of a consistent technological tradition, particularly notable when comparing different letters of the same sizes. For example, in the ascenders and descenders of 16 pt roman h, p, q, b, l, k and b (see Supplementary Figs. 1, 6 and 7 in Supplementary Materials 1) we generally see use of filing in these straight lines, and a combination of filing and engraving in the corners and angles. However, exceptions exist, like the straight line of letter l (made by a combination of filing and engraving) or the inner angle of the ascender of letter b (made by filing).

When compared to known modern examples, the Birmingham punches display remarkable artisanal skills. Although we can sometimes see small modifications of the design as the size of the letter is reduced (e.g. loss of curvatures, more obvious graver slips, or slight changes in the design of the pointed ends of letters, see Fig. 13 for some annotated examples and Supplementary Figs. 1 to 7 in Supplementary Materials 1), overall the quality of the lines of the Baskerville punches is exceptionally clean and smooth. This stands in contrast, for example, to modern 10 pt roman q, or modern 6 pt italic J. The quality of larger modern punches such as 16 pt italic Q, 40 pt roman V, or 12 pt roman E (see Supplementary Figs. 4 and 8 in Supplementary Materials 1), among others, seems neater than the smaller sizes. This might indicate a gradual loss over time of the high skills necessary to cut the smallest point sizes, for which the Birmingham workshop sustained very high standards of craftsmanship. Alternatively, the fact that Baskerville was not making his punches under commercial constraints (i.e. money and time available and no client to please) likely allowed him to take 5 years to implement the highest standards. Modern punch-cutters instead, worked under financial constraints, to a timeline and to client specifications. This might have impacted on the quality of the final product. Thus, they might still have a high degree of skills but time, client or financial pressures could have affected their optimum performance. Whatever the case, these apparent differences in quality should be corroborated through examination of a larger set of modern punches.

### Polishing the faces

**Fig. 14:**
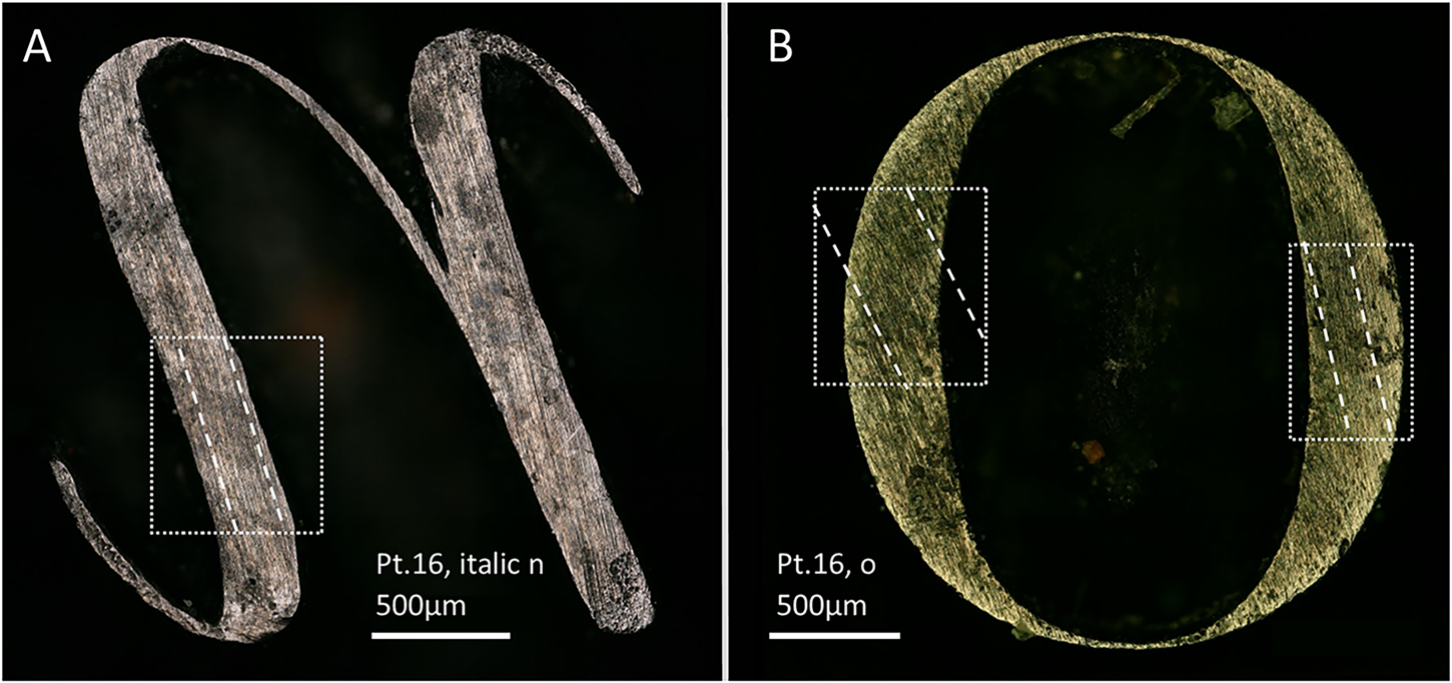
PPL and XPL micrographs of selected punches showing examples of polishing lines. **A** Polishing in a single direction. **B** Polishing lines in slightly different angles. The dotted lines indicate the direction of the polishing lines.

After letter-cutting, the faces were polished to a flat finish. According to the literature^11,21^, this process was undertaken by securing the punch in a holder, which facilitated polishing in a single direction, back and forth, with the punch face down on the abrading surface. In the *Manuel Typographique*^11^ and *Encyclopédie*^12^ an oilstone is mentioned for this process. Whatever the case, this operation would have left straight polishing lines in the direction of the polishing. Many of the Barkerville punches follow this model (Fig. 14A), with a single polishing direction documented in 37 out of the 64 punches analysed under the microscope, ranging from 60 pt down to 6 pt (see Supplementary Table 1 in Supplementary Materials 1).

In some letters with consistent polishing in a single direction, two polishing facets are visible, likely created accidentally when not holding the punch completely flat (Fig. 16A). This might indicate that polishing was indeed performed without a holder, at least in some cases. Furthermore, a few other punches (4 out of 64, see Supplementary Table 1 in Supplementary Materials 1) show the combination of different straight polishing directions (Fig. 14B), which may denote small unintentional rotations of the punch during the polishing stage. This, again, might be consistent with the absence of holders during the polishing process, or with a less secure holding of the punches.

**Fig. 15:**
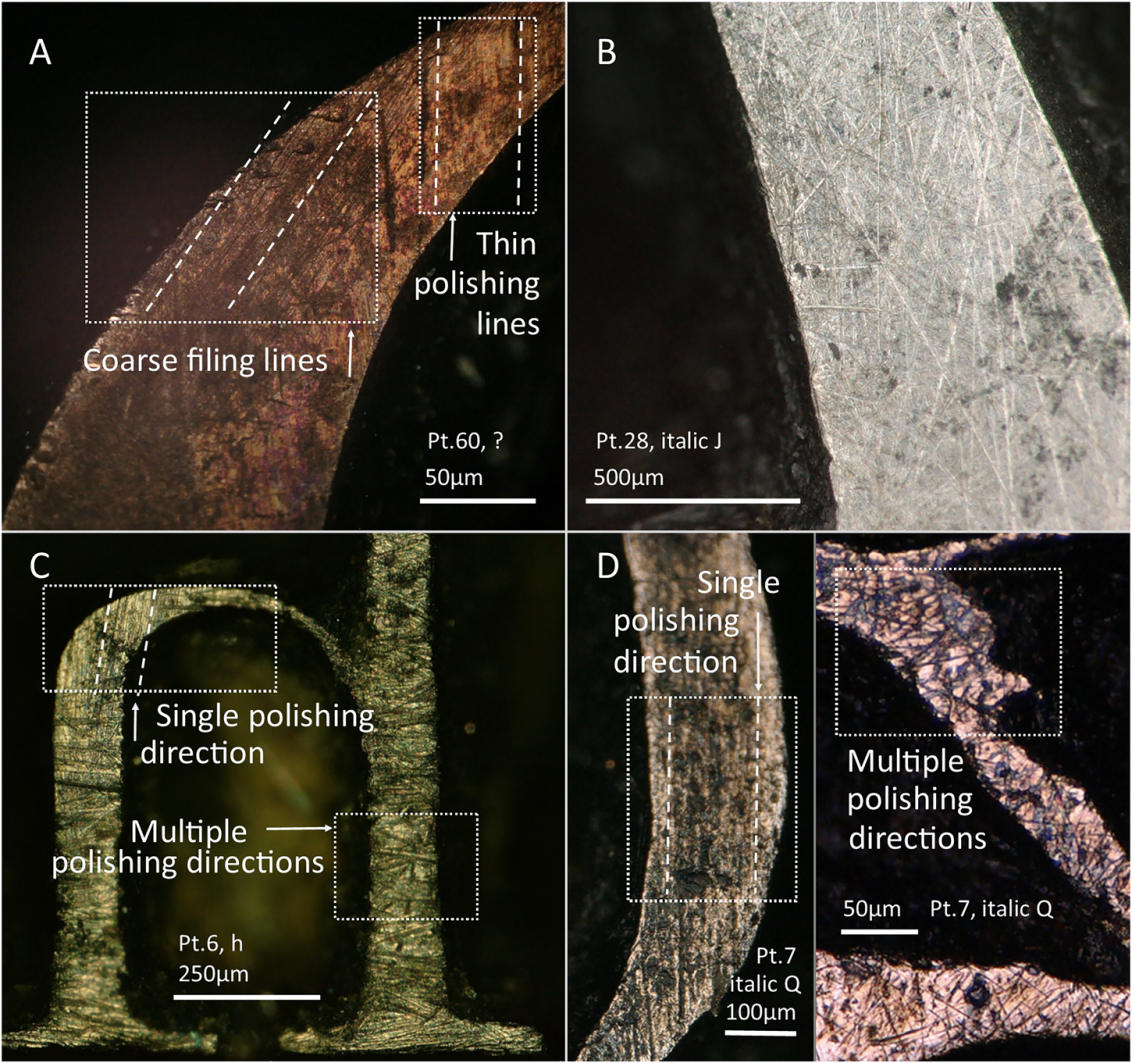
XPL and PPL micrographs of selected punches illustrating different polishing strategies. **A** Single polishing directions of different coarseness. **B** Multiple random polishing directions. **C**, **D** A combination of multiple random polishing directions with a single polishing direction in different areas of the face. The dotted lines indicate the direction of the polishing lines.

A separate group consists of punches that combine several polishing directions but of different coarseness (4 out of 64, see Supplementary Table 1 in Supplementary Materials 1). This would imply an initial polishing in a single direction and a later retouch with a coarser grit of a specific area of the punch in a different direction (Fig. 15A). This would be consistent with the use of a file after polishing to retouch the specific areas where the polishing result was not satisfactory.

In contrast, some punches (14 out of 64, see Supplementary Table 1 in Supplementary Materials 1) show several random directions of polishing coexisting in an area (Fig. 15B). This pattern is created when polishing in a circular motion, which promotes the random movement of the abrasive particles. These punches are concentrated in larger point sizes between 60 pt and 16 pt, with only one case in 8 pt and one in 6 pt. Therefore, this strategy is preferentially used for the largest point sizes. Other punches (4 out of 64, see Supplementary Table 1 in Supplementary Materials 1) have a combination of this pattern with areas where a single polishing direction is visible (Fig. 15C, D). These instances are difficult to explain but likely represent different cycles of polishing using different motions on different polishing surfaces. The sample, however, remains too small to draw further conclusions. Finally, it is also possible to find within the same punch the combination of areas with different directions of polishing, as well as separate areas with straight polishing lines in different directions, like 12 pt italic J, which is the only example of this kind. In this case, all the polishing directions seem of the same coarseness, which might imply a polishing process in which circular and vertical movements were combined, with uneven pressure.

Overall, and with some exceptions, the polishing strategy involved a vertical polishing motion for the smallest point sizes, and a circular polishing motion for the largest point ones. It makes sense that the circular motion was applied especially to the largest punches because as they have a larger surface to be polished flat, it is easier to maintain a flat surface when moving the hand in circles than when polishing back and forth, which in these cases could have created a more obvious facet at the area where more pressure is put with each new movement. Therefore, the application of polishing techniques was an informed choice. The rest of polishing patterns registered can be understood as exceptions to the general rule, in line with the versatility and adaptations observed at other manufacturing stages.

### Post-cutting processes: heat treatment, recutting and wear & tear

After punch-cutting, the objects were heat-treated to enhance the toughness of the steel. These treatments likely involved heating the whole punch—or part of it—and immediately quenching it in water or other liquid to release the heat quickly. These processes create changes in the microstructure of the metal that enhance its toughness^23^. External evidence of heat treatment are the changes in colour across the tip of some punches, ranging from blue/purple to yellow (Fig. 7). Although these are not visible in all punches, it is possible that the thin layer of corrosion covering most of the items is influencing our observations. Further micro-invasive research on these punches will clarify the specific heat treatment(s) used and how they interlink with the quality of the metal used.

After heat treatment, the punches were ready to be used and later stored. As we observed, many of the Birmingham punches were thoroughly covered by a sticky substance (see for instance the engraved angle in Fig. 11C), and we ran FTIR analyses on some of these residues. The substance of 40 pt roman h and 40 pt roman ? (i.e. question mark character) was consistent with bees wax, whereas that of 12 pt italic ffi had the closest resemblance to hexadecanediol notwithstanding some disagreements in some parts of the spectrum. This is a compound rarely found in its free form in nature but commonly derived from natural precursors such as fatty acids and alcohols present in coconut oil, palm oil and animal fats (see Supplementary Figs. 10 to 12 in Supplementary Materials 1). These analyses illustrate the use of different coatings to prevent corrosion during storage (see the Discussion section).

**Fig. 16:**
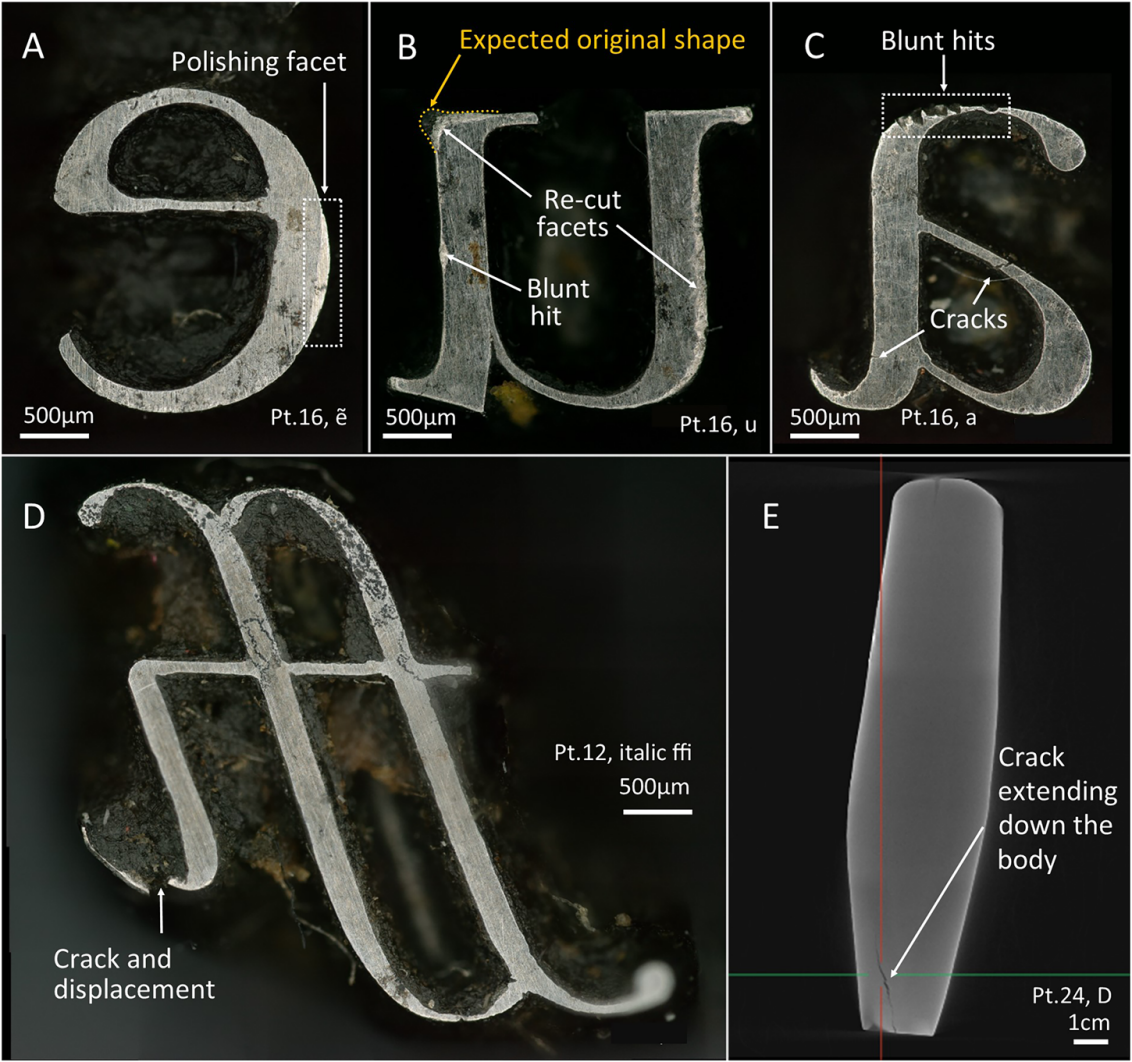
PPL micrographs and µCT cross-section of selected punches showing evidence of polishing facets, and wear and tear on the faces. **A** Polishing facet. **B** Evidence of recutting of the letter. **C, D, E** Evidence of wear and tear.

But the story that the witness marks on the punches can tell us does not end here. Based on historical accounts on the collection (see above), some punches were likely recut, both at Baskerville’s times and later on, to slightly modify their design. In the selection of punches studied, evidence of re-cutting was only observed in 16 pt roman u. In this punch, two different areas seem to have been modified by engraving, creating a very evident facet close to the face of the punch (Fig. 16B).

And finally, because of their prolonged use, the punches show strong wear and tear. Starting with the hammer ends, the Birmingham punches show two types of evidence of heavy use in this area: (1) cracks and/or (2) material displacements (Fig. 8). Cracks at the hammer ends are more common in the largest punches and, although created through heavy use, their development can be related to deficiencies in the heat-treatment process or to faults caused by undesirable slag inclusions or poorly consolidated metal. In most cases, the hammer end cracks do not appear to have prevented continued use of the punch, and occasional filing marks over cracks indicate a desire to amend the damage. However, the collection might be lacking punches that were discarded after more serious cracks.

Consecutive striking of the hammer ends also resulted in metal displacements at these areas (Fig. 8). When this material displacement was too pronounced to continue using the punch, the hammer end and its corners could be filed down to remove the displaced material. This seems to be the case of the 16 pt roman Q, whose filing marks extend down a single side of the body close to the hammer end, likely reflecting the side where material was displaced and removed through filing (Fig. 8). As a result, this punch is shorter than the rest of those of its point size (3.5 cm compared to the 3.9 cm median for 16 pt punches).

Finally, in one case it was possible to clearly see how an original face of a punch displaying a letter c was repurposed as a hammer end (Fig. 8). This might have happened after prolonged use of the c if cracks developed on the face, or perhaps it was discarded during the punch-cutting process if the progression of the design was not satisfactory. Whatever the case, the original face was filed down and repurposed as a hammer end, which again exemplifies the versatility of the *chaîne opératoire* used at the Baskerville workshop.

When looking at the wear and tear of the faces we can see blunt hits in corners (Fig. 16B, C) likely resulting from having dropped the punches or hit them accidentally against a hard surface. Other evidence is the disconnection or loss of parts of the punch face, likely due to repetitive use or sub-optimal heat treatment. For example, 12 pt italic ffi which has a clear displacement of the terminal of its i, would have likely been unusable after that (Fig. 16D). Interestingly, this particular punch was manufactured differently to others: all the terminals of the i’s of larger and smaller sizes were made through engraving, whereas the tail of 12 pt italic ffi was produced by filing (see Supplementary Fig. 2 in Supplementary Materials 1). This resulted in an extremely thin tail that likely facilitated breakage later during use. Thus, this particular case of wear and tear exemplifies how an adequate selection of the manufacture technique can have an important impact on the later performance and durability of the tool. Finally, it is also possible to see cracks on the faces of some punches that sometimes extend down the body of the punch (Fig. 16E).

## Discussion

**Fig. 17:**
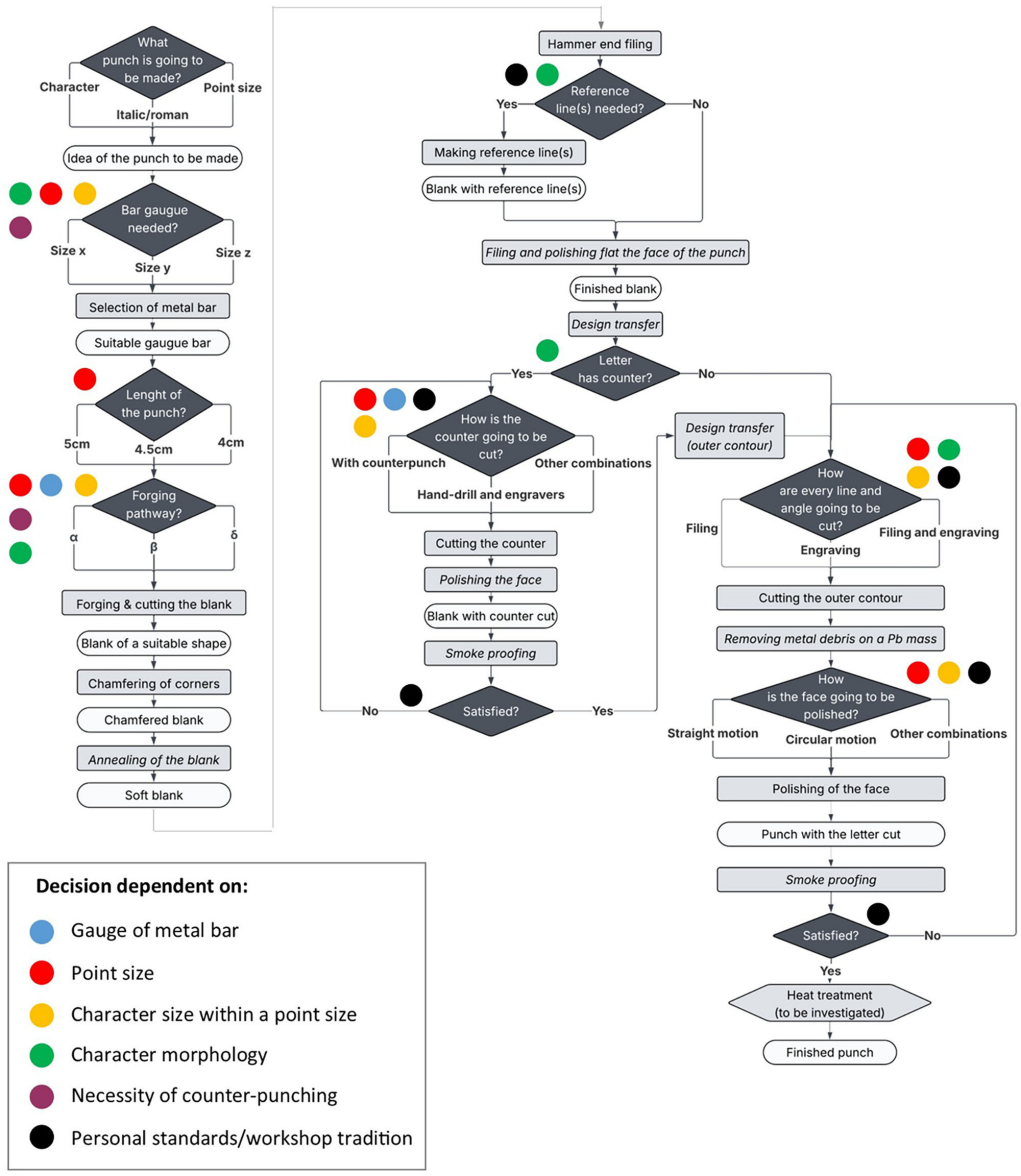
Simplified diagram of the *chaîne opératoire* of punch-making at the Baskerville workshop. Steps in italics leave no traces on the punches.

The study of the Baskerville punches using macroscopic observations, typology, stereo and digital microscopy, µCT, radiography and FTIR, coupled with reference to modern craft practice, has allowed us, for the first time, to reverse engineer the *chaîne opératoire* of the Baskerville Birmingham typographic punches. This is characterised by a set of consecutive tasks for which different technological solutions are often applied depending on specific variables. In this section we offer a detailed account of the *chaîne opératoire* used at the Birmingham workshop (Fig. 17). To do so, we combine the information gathered from the scientific analyses of the punches with 17^th^- and 18^th^-century historical accounts of punch-making. Beyond specifics of materials, tools and techniques, this approach ultimately results in a detailed account on the process of 18^th^-century punch-making and allows to better understand the dynamics of the Birmingham workshop and its technological tradition. This not only informs the broader context but also paves the way for future comparative studies.

The process started by deciding which punch was to be cut. This was an important decision, because the character morphology, its point size, size within a point size (i.e. a ! symbol and a capital Q of the same point size have different volumes) and whether the punch-maker was thinking of using a counterpunch, would affect the selection of a specific metal bar gauge. After selecting a bar of suitable dimensions, the process continued at the forge. There, the rod was worked following a specific forging pathway (α, β or δ) to modify the surface naturally provided by the bar to fit the desired letter. While metal bars of different gauges could be used to fit letters of broadly similar dimensions, the forging pathway selected allowed for finer adjustments to letter size even before the cutting started. Interestingly, only the α forging pathway is described in the *Manuel Typographique*^11^ so this study adds nuance into the variability of the forging processes in use.

Based on our observations, selecting the forging pathway was dependent on the point size of the letter, the character size within a point size (i.e. the space it occupied), as well as the gauge of the available metal bar. The *Mechanick Exercises* notes that if a letter was to be counterpunched, the metal surface available should be comparatively larger. Equally, for italics, the area of the blank close to the face was slightly compressed at the forge to save the punch-cutter from spending extra time removing more metal^6^. Thus, character morphology was likely also relevant for Baskerville, especially if further research is able to confirm the use of counterpunches.

During the forging stage, the original bar was sectioned in different lengths (around 5, 4.5 or 4 cm) depending on the point size to be cut, with longer blanks used for larger point sizes. Interestingly, the *Manuel Typographique*^11^ indicates that blanks were cut at about 2 inches (5.08 cm), corresponding to the largest punches in the Baskerville collection. However, our study indicates that other possibilities coexisted at the Birmingham workshop.

The dome-shaped hammer ends observed on the punches seem consistent with rod cutting on a hot cut hardy (Fig. 3). However, other techniques seem to have been traditionally used; for example, Moxon describes the use of a ‘*half-round file, or a cold-chissel*’ to section the iron bars^6^.

When the general shaping of the blank concluded, its corners were chamfered. Then, *Mechanick Exercises*^6^, the *Manuel Typographique*^11^ and the *Encyclopédie*^12^ agree that the finished blanks were annealed before cutting started, so they were easier to work. Annealing involved the heating of the steel blanks between 800–1200 °C to promote a change in the crystal structure of the material without altering its shape (i.e. producing austenite). When austenite slowly cools down, it results in the softest possible steel thanks to how its crystal structure settles at room temperature^24^. This is a crucial step, because it will precisely allow the use of steel tools (e.g. files and gravers) to cut the letters into the soft steel. This is because while the steel of the blank is in its softest possible state, the steel files and gravers have been heat-treated differently, producing a tougher steel able to work softer versions of the same material.

Fournier describes two ways of annealing the blanks: directly holding the blanks on the naked flame, or introducing them into closed crucibles full of ashes. In both cases the punches were heated and then left to cool down gently at the fireplace as the fire was extinguished. It is likely that a similar approach was used at the Birmingham workshop even if our current evidence cannot address this step of the *chaîne opératoire*. This operation denotes that, beyond mastering how to cut a letter, punch-makers had advanced understanding of the changing material properties of iron and steel when heat-treated.

Next, the hammer end was prepared with a general rough filing, as evidenced in the tool marks observed. Although an image in the *Manuel des Arts et Métiers* depicts a person roughly filing the blanks, a process that likely included the hammer end^8^, this step is only explicitly mentioned in the *Encyclopédie*^12^, where also the making of reference marks across the sides of the punches, such as the ‘signature’, is mentioned. These served to maintain the orientation of the punch consistent across the later stages of the process, so the character morphology was a relevant factor affecting the decision of including them. However, their inclusion is not always consistent. The key 17^th^- and 18^th^-century accounts agree that the next step would be the preparation of the face of the punch, by filing and polishing it flat, although practically no relevant witness marks remain on the Birmingham punches. The *Manuel Typographique*^11^ and the *Encyclopédie*^12^ indicate that files and an oilstone were used, and *Mechanick Exercises*^6^ also describes the process of face preparation in detail, involving several stages of filing. The Birmingham workshop likely used these methods.

Afterwards, it would be necessary to transfer the design of the letter onto the metal surface—a process for which practically no evidence is left in our collection except for two minor reference marks (Fig. 9). In Fournier’s description of this stage^11^ counterpunches were used as guiding marks for the counters, around which the letters were drawn using a bevel. In contrast, Moxon says that he ‘*draws or marks the exact shape of the letter, with a pen and ink if the letter be large, or with a smooth blunted point of a needle if it be small: Then, with sizable and proper shaped and pointed sculptors and gravers, digs or sculps out the steel between the stroaks or marks he made on the face of the punch, and leaves the marks standing on the face*’. The general absence of guiding marks in the Birmingham punches might indicate that the favoured approach at the Baskerville workshop may have involved ink as opposed to scraping the metal surface. Nonetheless, note that the later description by Moxon once again takes into account the size of the letter as a variable that conditioned the approach selected.

At this stage, if counterpunches were used (to be confirmed), it is likely that new rounds of filing and polishing flat the face of the punch were used (Fig. 17). If counterpunches were not used, then the inner counter of the letter was likely cut first using a drill and/or gravers. After this process, a new round of filing and polishing the face of the punch flat likely occurred, followed, if necessary, by a new design transfer of the outer contours of the letter. Of these stages we only have written accounts in the books already mentioned^6,11,12^, and no traces on the original objects remain.

Moving on to the shaping of the outer contours of letters, both the *Manuel Typographique*^11^ and *Mechanick Exercises*^6^ emphasise the use of filing for the external contours of letters, and Fournier describes the use of gravers for the external contours of punches with flower designs, followed by finishing touches with a knife-file. In the *Encyclopédie*^12^ it is also mentioned that it is possible to use both files and gravers to finish the letter contour. Modern accounts on punch-cutting, such as the descriptions provided by Smeijers^22^ also emphasise the combination of filing with engraving (generally in line with one of the strategies followed at the Birmingham workshop) to shape the outer contour of the letters. These descriptions already illustrate that both filing and engraving were considered depending on the design to be cut and the personal preferences of the craftsperson. This fits our observations under the microscope, even if we were able to obtain further nuance on how both filing and engraving were combined.

Punch-cutters at the Birmingham workshop combined three different approaches for every angle and line they carved in the outer contours of the letters: the sole reliance on filing, the combination of filing and engraving, or the sole reliance on engraving. Their choices seem to be conditioned once again by the point size (with larger letters favouring filing and smaller ones engraving), but also by the letter morphology (i.e. their acute angles or rounded external contours). It is also likely that personal/workshop standards also affected this decision, as for example the apparent favouring of filing in the Birmingham punches contrasts with a more general use of engraving in the few modern punches studied.

After the letter was cut, it is possible that the punch was struck against a lead mass to mechanically remove remaining metal debris. This step is described by Fournier^11^ and depicted in the plates of the *Manuel des Arts et Métiers*^8^, but it would leave no recognisable trace on the punches. Then, the punch-cutter had to choose a strategy for the last polishing of the face. According to our study, smaller punches were polished in a single straight direction, whereas larger ones were polished in multiple directions, probably using rotary motion. Other possibilities that implied multiple polishing rounds or a final retouching with a rougher file were also identified, perhaps reflecting personal preferences within the general workshop culture, or *ad hoc* adaptations to how the material was responding. This level of detail in the understanding of the polishing stage was not described in any of the written sources available.

A fundamental step that is invisible in the extant punches was smoke-proofing. Smoke-proofing allowed the punch-cutter to regularly check the shape of the letter while working on it, and at the end of the polishing process. No toolmarks remain from this process, but the process is depicted, for example, at the *Manuel des Arts et Métiers*^8^, as well as described in the *Manuel Typographique*^11^. Smoke-proofing consists of covering the freshly cut character with a thin layer of soot from a candle or oil lamp, blackening the surface. Then, the punch is pressed against paper to leave an impression of the character, which is examined to assess whether further corrections are needed on the punch. If so, the punch-cutter would proceed again to further engrave or cut metal, then again to the polishing and again to smoke-proofing (Fig. 17). Only when the punch-maker was satisfied with the result would they proceed to the heat treatment stage.

Based on the observed changes in colour at the tip of some of the punches (Fig. 7), it is inferred that punches were heat treated to enhance the material properties of the metal. Although the processes of heat treatment can only be disentangled through invasive research that goes beyond the scope of this paper, it is also very likely that different approaches towards quenching and tempering coexisted. Whatever the case, after heat-treatment, the punch was likely cleaned, before applying a protective coating. Fournier describes coating the punches with olive oil to prevent corrosion while stored^11^. Most of the Birmingham punches also remain coated by a dark sticky substance. This was characterised as bees wax in some instances and as an animal/plant-based undetermined fat in others. This indicates that variation also existed in this coating treatment, although it is impossible to know if these analysed substances were applied during Baskerville’s time or later.

While broadly recognisable as a coherent set of practices, the *chaîne opératoire* of the Birmingham punches is characterised by remarkable versatility at multiple stages of manufacturing. Crucially, the variability recorded in this study surpasses that described in the relatively contemporaneous text sources. This evidences that the information recorded in written accounts does not always correspond to reality and that material culture analyses are necessary for an accurate understanding of the past in general, and of past technologies in particular^25^. At this point, it is necessary to note that Baskerville was an amateur when he started crafting punches. Contrary to the modern punch-cutters that contributed to the collection later in time, he learnt this craft by himself. This lack of apprenticeship might have contributed to him finding his own ways through the process of manufacture instead of replicating a master’s way of doing things.

Figure 17 summarises the key stages when it was necessary to choose between different alternatives, as well as the factors conditioning the choices. Variability can be explained with reference to six variables: gauge of the original metal bar, point size, character size within a point size, character morphology, necessity of using counterpunches, and personal standards/workshop tradition. Except for the last one, which represents the agency of the craftsperson, all these variables are primarily conditioned by artefact performance factors; i.e. factors related to the behavioural capabilities that the punch should possess at the end of the manufacturing process to fulfil its function^18^. However, the last variable, even if broadly defined, is as important as the rest in helping us explain the punches as they stand.

Assuming that only a few individuals were involved in the manufacture of these objects, one might expect a more consistent use of manufacturing recipes. However, while it is possible to define general rules (e.g. ‘large point sizes are cut preferentially by filing’ or ‘small point sizes are generally polished in a single direction’), exceptions and deviations are frequent. These general rules correspond to what studies of technology refer to as ‘knowing what’ (*connaissances*), a conscious understanding in practical terms of the logic behind a selected technological action^26,27^. By contrast, the frequent deviations from these general rules denote an ever-present tacit, kinaesthetic knowledge or know-how (*savoir faire*), that relies on intuition, embodied gestures and previous experiential practice^26–29^. The capacity of the punch-makers to move between the ‘knowing-what’ and ‘knowing-how’ is the driver of variability within our collection and technological tradition, reflecting the constant adaptability and implementation of contingency measures as the technological process developed. Such adaptability requires a high level of skill, at odds with strict recipe books but crucial to achieve the high-performance standards of the Birmingham workshop. Conversely, this variability makes it practically impossible to identify idiosyncratic witness marks that might point to a batch of punches made by the same individual. While this has been attempted in other collections^22^, it would seem challenging for the Birmingham punches.

Having said this, the combination of the general patterns of behaviour observed—as summarised in the Results section—allows us to define the technological tradition followed at the Birmingham workshop. Besides the versatility and technological choices already mentioned, key aspects of this tradition include: (1) the tight integration of forging in the punch-making process, (2) the great care in removing reference marks left on the faces of the punches, (3) the favouring of filing for as long as possible, combined with engraving only when necessary, (4) the incorporation of engraving to define the outer contours of the letters for the smallest point sizes and difficult-to-access angles, (5) the high quality of the finish lines, (6) the flexible approaches towards the final polishing of the faces. Future studies should explore whether this technological tradition was developed at the Baskerville workshop or inherited/transmitted from earlier artisans.

This tradition stands in contrast to what we can observe from the ten modern punches analysed for this paper, manufactured in the 19^th^ and 20^th^ centuries. Some of the key differences with the Birmingham tradition are: absence of forging of the blanks, different strategy for transferring the design of the letter with more reference guide marks left behind, higher reliance on engraving, potential lower personal/workshop standards of quality, and lesser variability in polishing strategies of the faces.

We acknowledge that the small number of modern punches studied include the work of different known and unknown manufacturers involved (e.g. 16 pt ß was made by Deberny and Peignot, and the step-punch 12 pt E by Fevrier according to the initials carved in them). Only further research will be able to offer more nuance and perhaps identify distinct technological traditions among the modern punches. Moreover, it will be necessary to assess other collections contemporaneous to the Birmingham punches, and earlier. This will allow to better document and understand changes in workshop practices in time and space, and better define technological traditions as workshop-specific, time-specific, or both. This is a common approach in archaeological studies of technology^30–38^. At the moment, lacking comparable studies, we remain ignorant about the idiosyncrasy of the Baskerville—and particularly the Birmingham—collection.

The workshop dynamics revealed by the Birmingham *chaîne opératoire* have important implications regarding terminology. On the basis of our study, we propose that ‘punch-making’ is a broader and more appropriate term, as opposed to the more common ‘punch-cutting’. This is because, for the 18^th^ century at least, a large part of the process of punch manufacture occurred at the forge. This realisation prompts two important observations.

The first is that we should be more alert to the complexity of the planning process. Given the identified entanglement between the forging pathway, and the point size and morphology of the character to be cut, integration between forging and cutting would be crucial: the blacksmith needed to know in advance what was required at the punch-cutter’s bench. *Mechanick Exercises*^6^ already emphasises the importance of this relationship between the forging stage and the final product, and the plates of the *Manuel des Arts et Métiers*^8^ depict forging in the same space as counterpunch-cutting. The required coordination between stages, added to the need to order metal bars of different gauges for different point sizes, considerably complicated the planning strategy as well as the flux of information within the workshop.

The second point is the question of whether blacksmith and punch-cutter were the same or different people within the Birmingham workshop. Blacksmiths usually make their own tools. These include things like chisels, but also punches. Thus, being able to produce blanks of these tools is a fundamental skill that any blacksmith would have been able to perform. For typographic punches in particular, in *Mechanick Exercises*^6^, Moxon says that ‘*The Letter-Cutter does either forge his steel-Punches, or procures them to be forged*’. This shows that forging skills could be available within the punch-making workshop. Although we do not have direct evidence, it is worth remembering the technical flexibility of John Baskerville himself, a craftsperson who easily moved between and across crafts (e.g. writing, stone cutting, japanning, book binding, paper making, etc^3^.), so it is plausible that he could have forged his own punches too. This would have facilitated both planning and the feedback loop between making and adjusting blanks and punches—though of course at the cost of even broader skills.

Finally, turning to the modern punches, the absence of forging indicates a complete restructurisation of the general dynamics of later printing workshops. The disappearance of the forge was most likely prompted by the availability of high-quality cold-rolled metal bars for punch manufacture, which could be filed and polished directly by the punch-cutter without further shape adjustments. Cold-rolling was successfully introduced in the late 18^th^ century^39^, which agrees with the expected 19^th^- and 20^th^-century chronologies for our modern additions. This episode of the history of printing technology illustrates how changes in the metal-making industry were directly affecting the organisation of labour in a printing workshop, emphasising the relationship between technology and society^40–44^. At some point in the 19^th^ century, therefore, punch-making becomes punch-cutting, as the forging stage is removed from the *chaîne opératoire* and the technological tradition. The exact moment when this happened is yet to be determined.

In summary, in this paper we presented the first analytical study of a collection of typographic punches: those manufactured by John Baskerville. Through a combination of archaeological science techniques and contemporaneous historical sources, and with reference to past and present craft practice, we reverse engineered the *chaîne opératoire* of punch-making at the Birmingham workshop, defined the broader technological tradition and explored the logic behind the technological choices at multiple stages of the process. In turn, this informed broader issues such as the skillset required, the materials and tools used and the links between punch-making and other industries and crafts, such as steel production, blacksmithing and metal engraving.

Superficially, the Birmingham punches appear highly standardised and they broadly reflect a shared technological tradition. However, our study reveals that, behind the quality and consistency of the finished letters that would make Baskerville’s books famous, there was remarkable variability in punch-making. This was particularly noticeable in the skilled application of contingency measures in response to the behaviour of the materials in order to achieve the desired standards. Most likely, embodied knowledge allowed artisans to move flexibly between ‘knowing what’ and ‘knowing how’. Further, our analyses demonstrated a tight interconnection among all the stages of the *chaîne opératoire*, working backwards in a decision tree that started with the planned character to be cut. This variability was not accounted for in contemporaneous textual records.

The proposed *chaîne opératoire* for 18^th^-century punches, which places great importance to the forging stage, makes us favour the term ‘punch-making’ to refer to this craft during this time. This term encourages us to reflect on the necessary coordination between the planning and manufacturing stages within a workshop. It is only at an unspecified moment between the 18^th^ and 19^th^ century, when punch-making turns into punch-cutting as the forging stage is removed from common practice in workshops. We therefore encourage scholars to consider using the term punch-making as opposed to punch-cutting when relevant in order to account for the whole process of manufacturing beyond the cutting of the letter.

Overall, this study offers a new perspective on 18^th^-century punch-making, as well as a non-destructive methodological approach applicable to other collections. While the *chaîne opératoire* framework and the focus on material culture are well-established in archaeology and anthropology, we contend that it still has much to offer to printing history and to science and technology studies more generally. This is particularly important and useful when dealing with an endangered craft such as punch-making. Future comparative studies on similar materials will allow us to extend and compare our observations across time and space, enabling a history of craft practice that deservedly places materials and craftspeople at the centre.

## Supplementary information

Supplementary materials 1

Supplementary materials 2

## Data Availability

We confirm that all data supporting this study are available in the manuscript and in the Supplementary Materials related to it.
