# Supplementary material for: The manufacture of the Baskerville typographic punches: the versatile chaîne opératoire of an 18th-century printing workshop
Source: NPJ Herit Sci. 2026 Apr 14;14(1):246. doi: 10.1038/s40494-026-02504-9 (PMC13079102; doi:10.1038/s40494-026-02504-9)
Supplement: Supplementary file 1 — Supplementary materials 1 [file 40494_2026_2504_MOESM1_ESM.pdf]

Supplementary Materials 1: Detailed analytical data

The manufacture of the Baskerville typographic punches: the versatile *chaîne opératoire* of an 18th century printing workshop

Julia Montes-Landa; Mark Box; Caroline Archer-Parré; Ann-Marie Carey; Maciej Pawlikowski; Marcos Martinón-Torres

Supplementary Table 1. Summary of results of the 64 selected punches for which a detailed examination was conducted.

CA no.	Punch code	Pt. size	Length (mm)	Thickness (mm)	Length (cm)	Thickness (rounded, mm)	Forging pathway	Polishing	Notes	µCT	X-Rays	FTIR
CA240040	Q	Pt. 60	45.5	15.6x18.9	4.6	19x16	α	Several random directions				
CA240114	g	Pt. 60	48.9	13.6x10.4	4.9	14x10	α	Single direction				
CA240111	h	Pt. 60	50.9	14.5x12.4	5.1	15x12	α	Single direction		x	x	
CA240112	?	Pt. 60	46.5	13.3x12	4.7	13x12	α	Different straight polishing directions (different coarseness)				
CA240113	6	Pt. 60	57.7	17.7x16.7	5.8	18x17	α	Single direction				
CA240116	Italic J	Pt. 40	48.7	12.3x10.6	4.9	12x11	β	Several random directions				
CA240115	Italic ffi	Pt. 40	50.3	14.4x11.3	5.0	14x11	β	Several random directions				
CA240033	V	Pt. 40	58.2	15x16.4	5.8	15x16	Not forged	Single direction	MODERN			
CA240118	?	Pt. 40	36.2	8.1x6.4	3.6	8x6	α	Different straight polishing directions (different coarseness)				x
CA240119	6	Pt. 40	44.4	9.2x6.2	4.4	9x6	α	Several random directions		x	x	
CA240117	h	Pt. 40	48.8	11.6x9.9	4.9	12x10	β	Several random directions				x
CA240122	Q	Pt. 28	50.3	11.2x10.5	5.0	11x11	β	Single direction				
CA240121	Italic J	Pt. 28	50.2	8.3x7.3	5.0	8x7	α	Several random directions				

CA240120	Italic ffi	Pt. 28	46.6	10.9x9.1	4.7	11x9	α	Several random directions				
CA250014	D	Pt.24	33.6	10.0x9.5	4.6	10x10	β	Not analysed		x	x	
CA240041	Q	Pt. 20 (Peig.)	45.6	8.7x9.5	4.6	10x9	β	Single direction	Peignot box*but Birmingham			
CA240034	Q	Pt. 16	35.4	7.2x6.5	3.5	7x7	α	Single direction + several random directions	Reworking of shank			
CA240141	a	Pt. 16	40.7	5.1x4.2	4.1	5x4	δ	Several random directions				
CA240140	c	Pt. 16	40.1	4.7x4.2	4.0	5x4	β	Single direction				
CA240139	e	Pt. 16	44.1	4.5x4.4	4.4	5x4	β	Single direction				
CA240133	q	Pt. 16	39.4	5.3x4.4	3.9	5x4	δ	Several random directions				
CA240137	o	Pt. 16	40.1	4.8x 4.1	4.0	5x4	β	Different straight directions (same coarseness)				
CA240129	k	Pt. 16	42.8	4.5x4.3	4.3	5x4	α	Single direction				
CA240037	b	Pt. 16	39.9	5.9x5.5	4.0	6x6	δ	Several random directions				
CA240130	u	Pt. 16	41.3	4x5	4.1	5x4	δ	Single direction	Re-cut letter	x	x	
CA240138	i	Pt. 16	34.6	4.9x4.1	3.5	5x4	β	Single direction				
CA240136	n	Pt. 16	42.5	5.6x4.9	4.3	6x5	δ	Single direction				
CA240127	italic n	Pt. 16	38.5	4.2x4	3.8	4x4	β	Single direction				
CA240134	p	Pt. 16	36.1	4.8x4.7	3.6	5x5	δ	Single direction				
CA240126	l	Pt. 16	40.9	5.2x4.8	4.1	5x5	β	Single direction				
CA240142	f	Pt. 16	39.9	5x4.4	4.0	5x4	δ	Single direction				
CA240125	h	Pt. 16	42.2	5.6x4.9	4.2	6x5	β	Several random directions				
CA240135	è	Pt. 16	39.3	4.5x4.3	3.9	5x4	δ	Several random directions				
CA240131	ê	Pt. 16	39.7	5x4.3	4.0	5x4	β	Single direction				
CA240132	ë	Pt. 16	38.7	4.4x4.1	4.0	4x4	β	Single direction		x	x	
CA240128	~e	Pt. 16	42.7	4.8x4.2	4.3	5x4	δ	Single direction		x	x	
CA240030	Italic q	Pt. 16	38.3	5.6x5	3.8	6x5	β	Different straight polishing directions (different coarseness)				
CA240123	Italic ffi	Pt. 16	41.6	7.1x5.7	4.2	7x6	α	Single direction				

CA240124	Italic J	Pt. 16	38.1	5.1x4.8	3.8	5x5	α	Different straight directions (same coarseness)				
CA240031	Italic Q	Pt. 16	56.3	7.9x7.8	5.6	8x8	Not forged	Single direction	MODERN			
CA240035	ß	Pt. 16	58.5	8.3x8.1	5.9	8x8	Not forged	Single direction	MODERN – D&P carved	x	x	
CA240147	k	Pt. 12	35.2	3.7x3.2	3.5	4x3	β	Different straight polishing directions (different coarseness)				
CA240148	?	Pt. 12	38.1	3.7x3.5	3.8	4x4	δ	Single direction				
CA240145	Italic J	Pt. 12	40.6	5x5.6	4.1	6x5	δ	Several random directions + different straight polishing directions (same coarseness)				
CA240144	Italic ffi	Pt. 12	43.5	5.1x4.6	4.4	5x5	α	Single direction				x
CA240149	6	Pt. 12	45.8	6x6	4.6	6x6	Not forged	Single direction	MODERN			
CA240143	h	Pt. 12	45.5	5.7x6	4.6	6x6	Not forged	Single direction	MODERN			
CA240042	E step	Pt. 12	58.4	8x8	5.8	8x8	Not forged	Single direction	MODERN – FEVRIER carved	x	x	
CA240146	Q	Pt. 12	40.7	4.9x5.9	4.1	6x5	δ	Single direction				
CA240038	q	Pt. 10 (Peig)	44.5	5.2x5.5	4.5	6x5	Not forged	Single direction	MODERN – Peignot box*			
CA240152	Q	Pt. 8	39.1	4.3x3.8	3.9	4x4	δ	Single direction				
CA240150	italic ffi	Pt. 8	38.6	4.1x3.6	3.9	4x4	δ	Single direction + several random directions				
CA240154	?	Pt. 8	38.4	4.3x3.9	3.8	4x4	δ	Single direction				
CA240153	h	Pt. 8	41.6	3.4x3.0	4.2	3x3	β	Different straight directions (same coarseness)				
CA240151	Italic J	Pt. 8	39.3	3.6x3.6	3.9	4x4	β	Several random directions				

CA240039	Italic Q	Pt.7	39.2	3.8x3.1	3.9	4x3	δ	Single direction + several random directions		x	x	
CA240248	g	Pt. 6	41.3	3.4x3.3	4.1	3x3	δ	Several random directions				
CA240157	H	Pt. 6	38.7	3x2.9	3.9	3x3	δ	Single direction + several random directions				
CA240159	Italic g	Pt. 6	43.5	2.9x2.9	4.4	3x3	δ	Single direction				
CA240158	6	Pt. 6	42.1	3.1x2.8	4.2	3x3	δ	Different straight directions (same coarseness)				
CA240155	italic ffi	Pt. 6	42.5	3.5x3	4.3	4x3	δ	Single direction				
CA240032	Italic Q	Pt. 6	46.5	6.1x5.9	4.7	6x6	Not forged	Single direction	MODERN			
CA240036	Q	Pt. 6	41.8	5x5	4.2	5x5	β	Single direction		x	x	
CA240156	Italic J	Pt. 6	44.8	6x6.2	4.5	6x6	Not forged	Single direction	MODERN			
CA240043	Q	Unkown	53.4	6.6x6.6	5.3	6.6x6.6	Not forged	Single direction	MODERN – Peignot box*			

*The Peignot box: In March 1953 Charles Peignot wished to launch a new series of Baskerville types for which he needed to make fresh matrices. To do so, he requested 380 of the punches given to Cambridge—both roman and italic and in sizes from 6pt to 40pt—be returned to Paris on loan for close study. Some of these punches were given back to Cambridge in 1960, but a letter from Charles Peignot to John Dreyfus, dated 27 June 1973, shows that some remained in Paris. The Peignot box refers therefore to the box of punches returned by Peignot in the 1970s. Although it mostly contains modern duplicates, some punches look 18th century Birmingham objects.

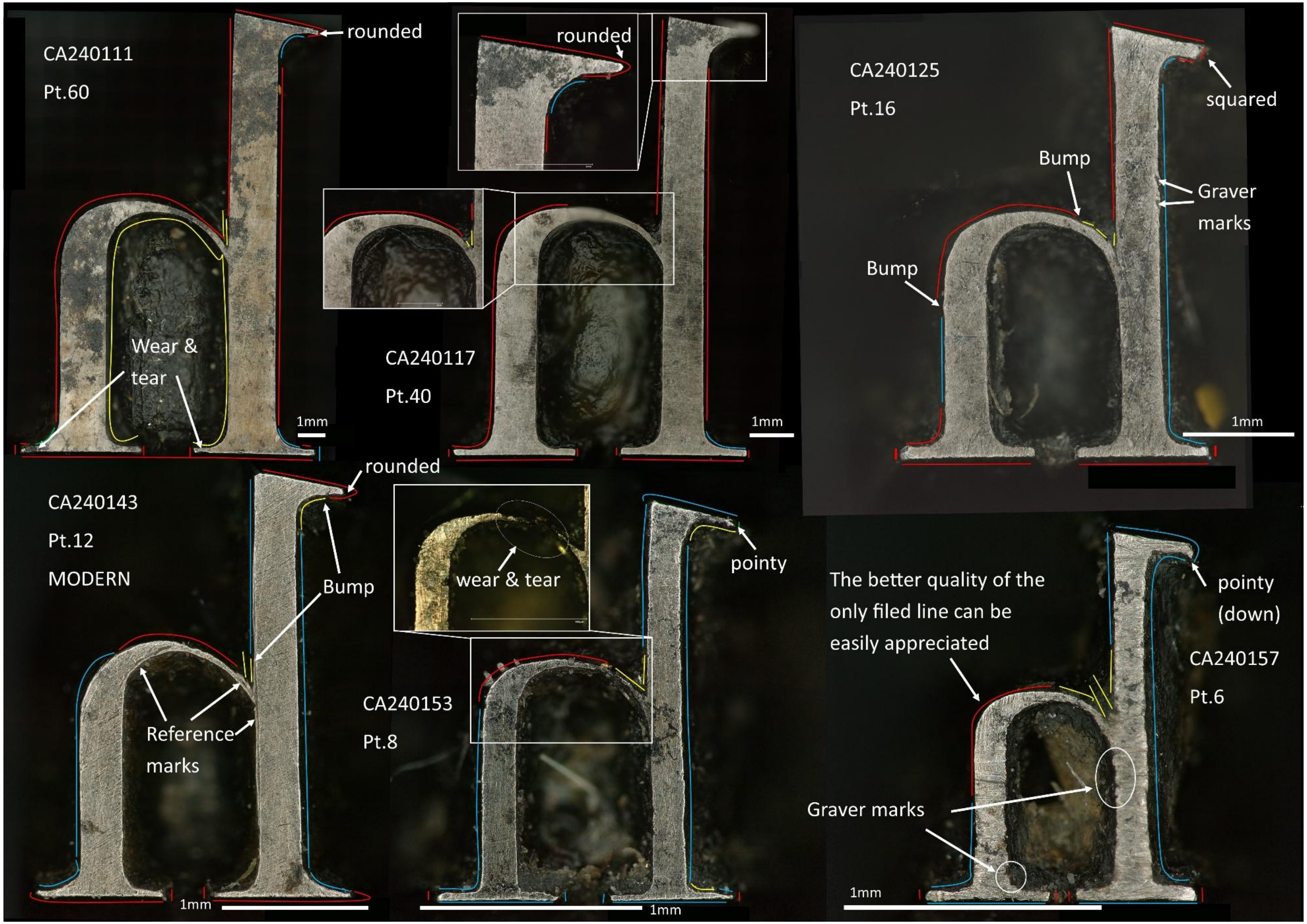

Supplementary Figure 1. Comparison of the techniques used to cut selected roman h's of different point sizes. Red lines indicate areas of the punch cut by filing, yellow lines indicate areas of the punch cut by engraving, and blue lines indicate areas of the punch cut by a combination of filing and engraving.

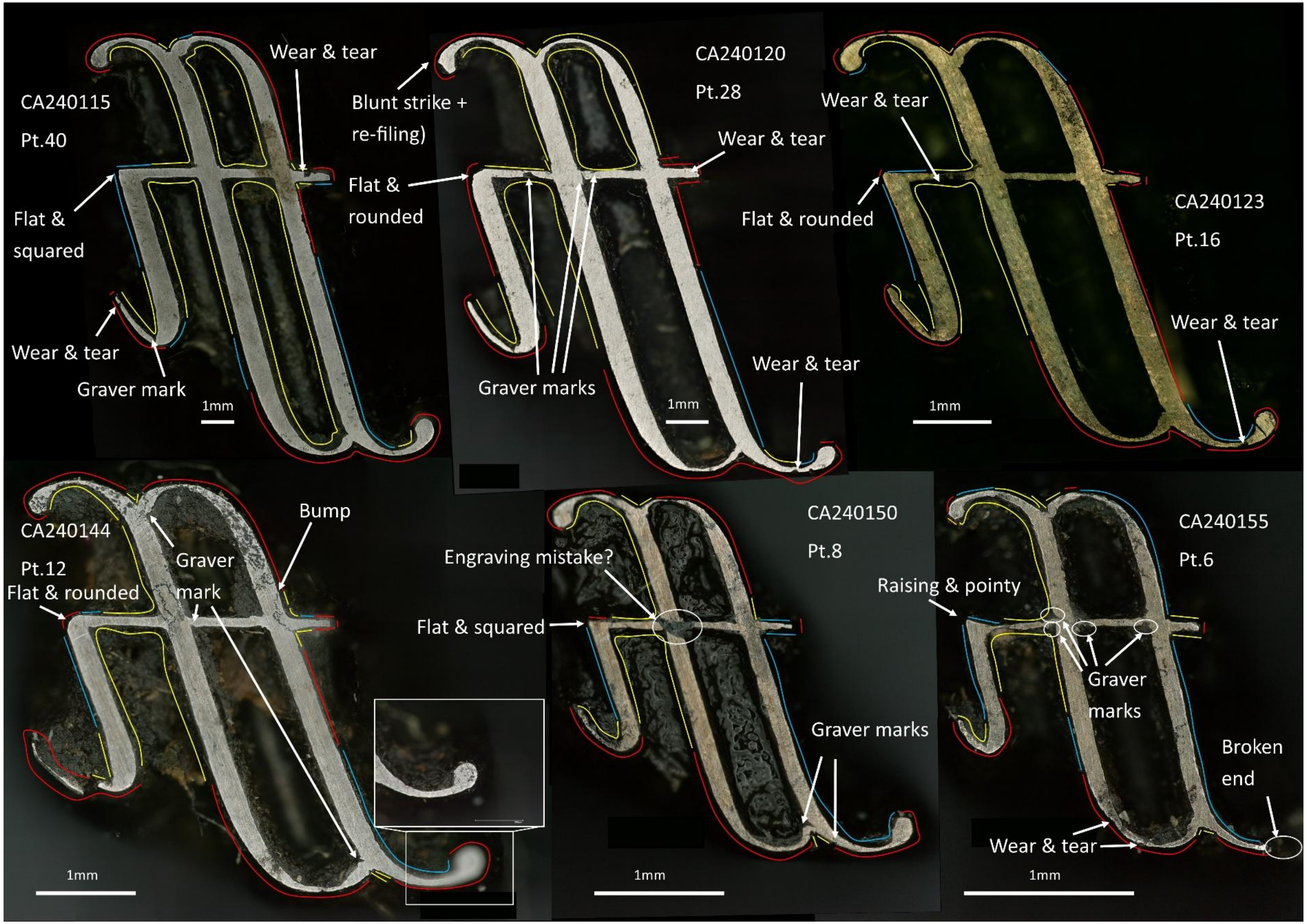

Supplementary Figure 2. Comparison of the techniques used to cut selected italic ffi's of different point sizes. Red lines indicate areas of the punch cut by filing, yellow lines indicate areas of the punch cut by engraving, and blue lines indicate areas of the punch cut by a combination of filing and engraving.

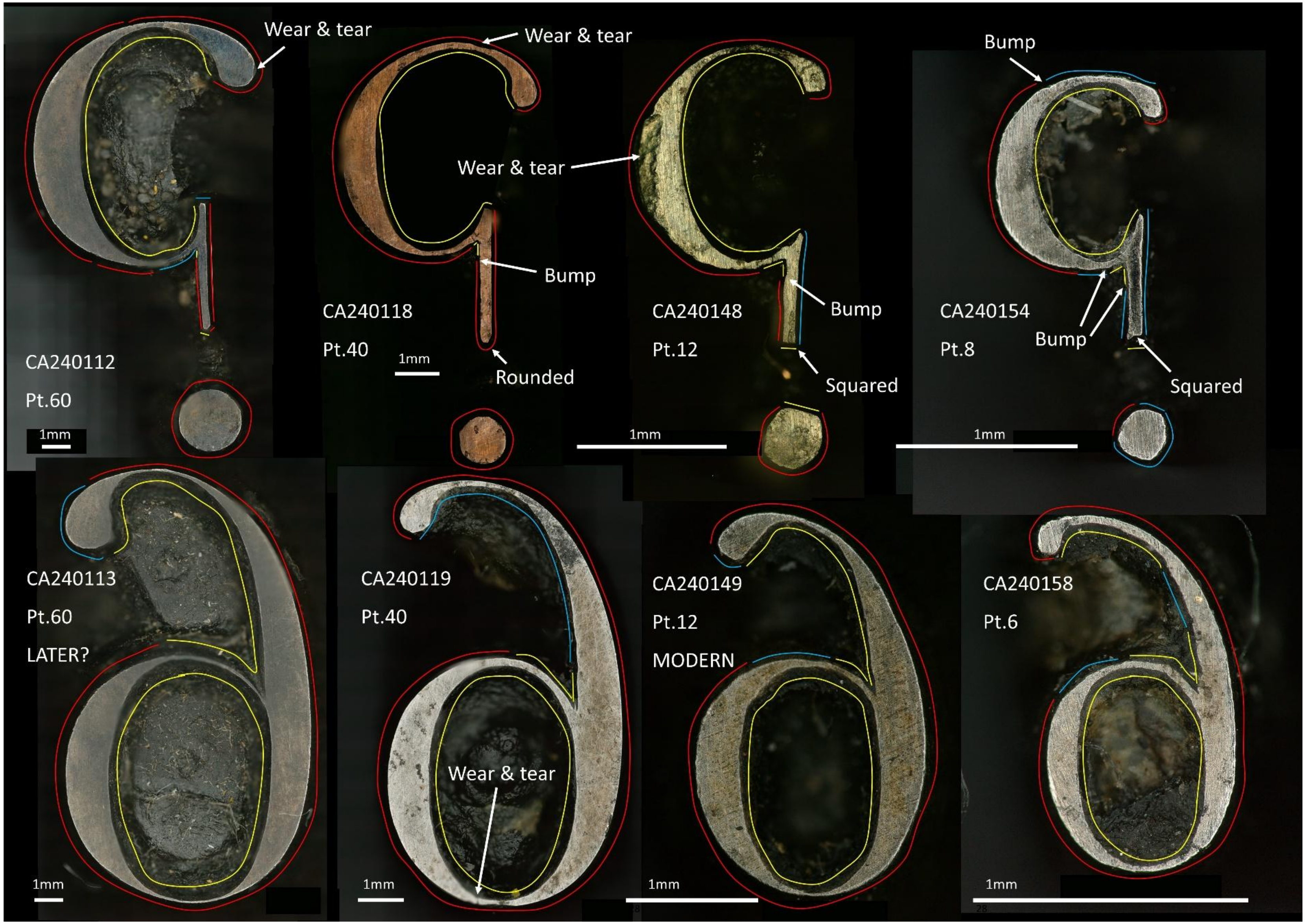

Supplementary Figure 3. Comparison of the techniques used to cut selected roman ?'s and 6's of different point sizes. Red lines indicate areas of the punch cut by filing, yellow lines indicate areas of the punch cut by engraving, and blue lines indicate areas of the punch cut by a combination of filing and engraving.

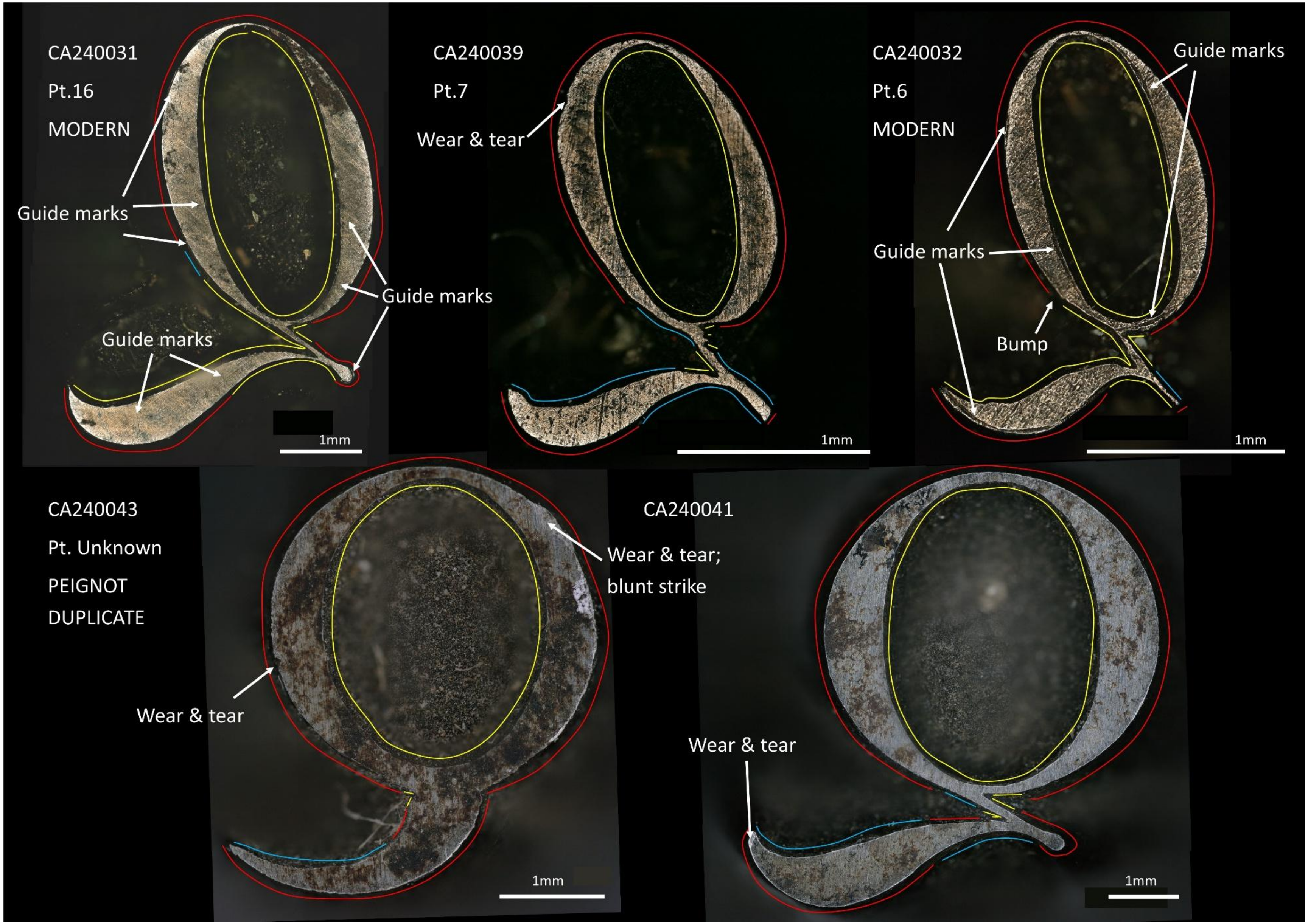

Supplementary Figure 4. Comparison of the techniques used to cut selected roman and italic Q's of different point sizes. Red lines indicate areas of the punch cut by filing, yellow lines indicate areas of the punch cut by engraving, and blue lines indicate areas of the punch cut by a combination of filing and engraving.

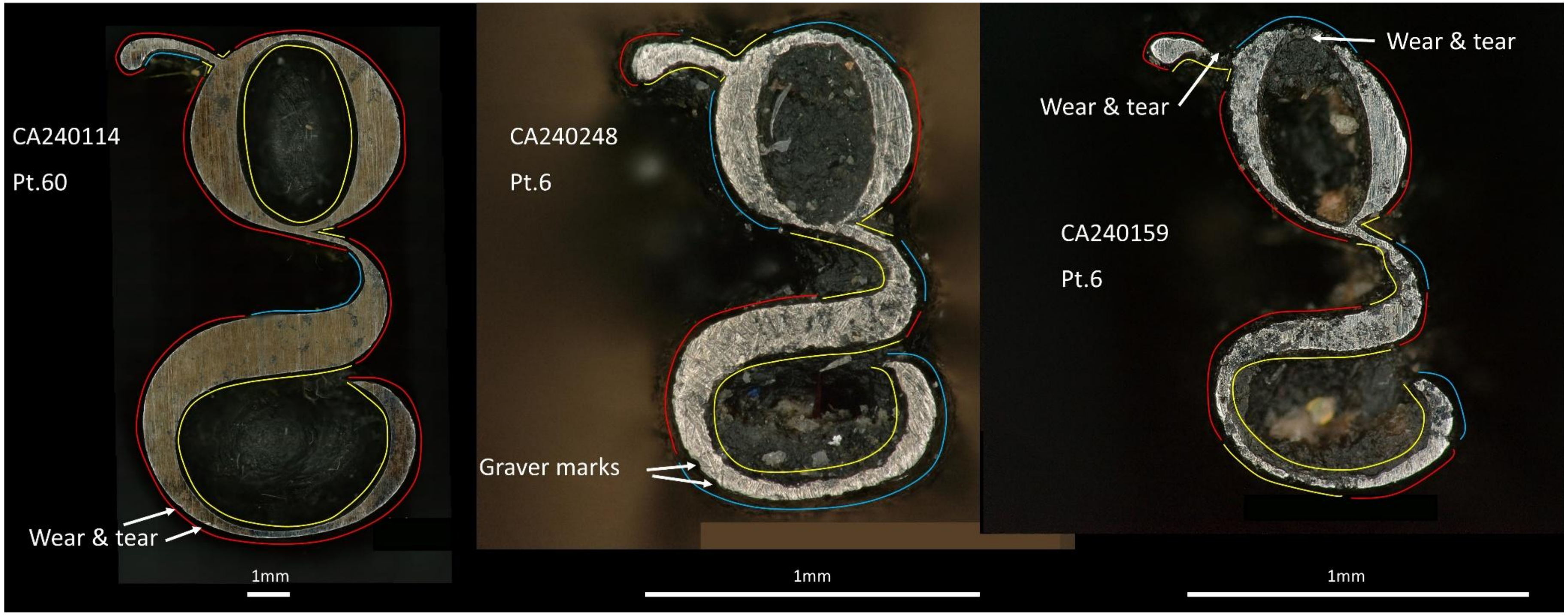

Supplementary Figure 5. Comparison of the techniques used to cut selected roman and italic g's of different point sizes. Red lines indicate areas of the punch cut by filing, yellow lines indicate areas of the punch cut by engraving, and blue lines indicate areas of the punch cut by a combination of filing and engraving.

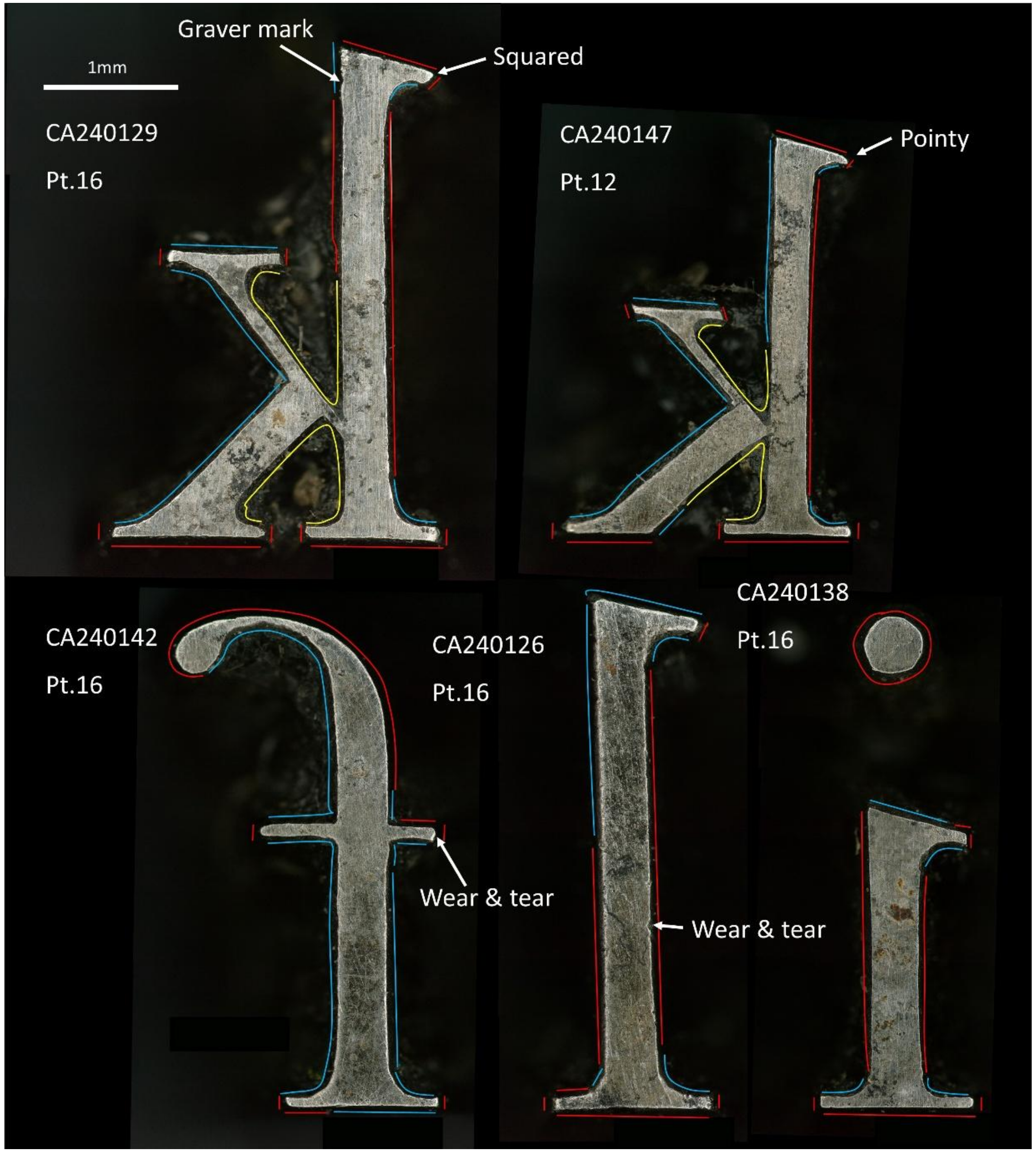

Supplementary Figure 6. Comparison of the techniques used to cut pt. 16 roman k, f, l and i, and pt. 12 roman k. Red lines indicate areas of the punch cut by filing, yellow lines indicate areas of the punch cut by engraving, and blue lines indicate areas of the punch cut by a combination of filing and engraving.

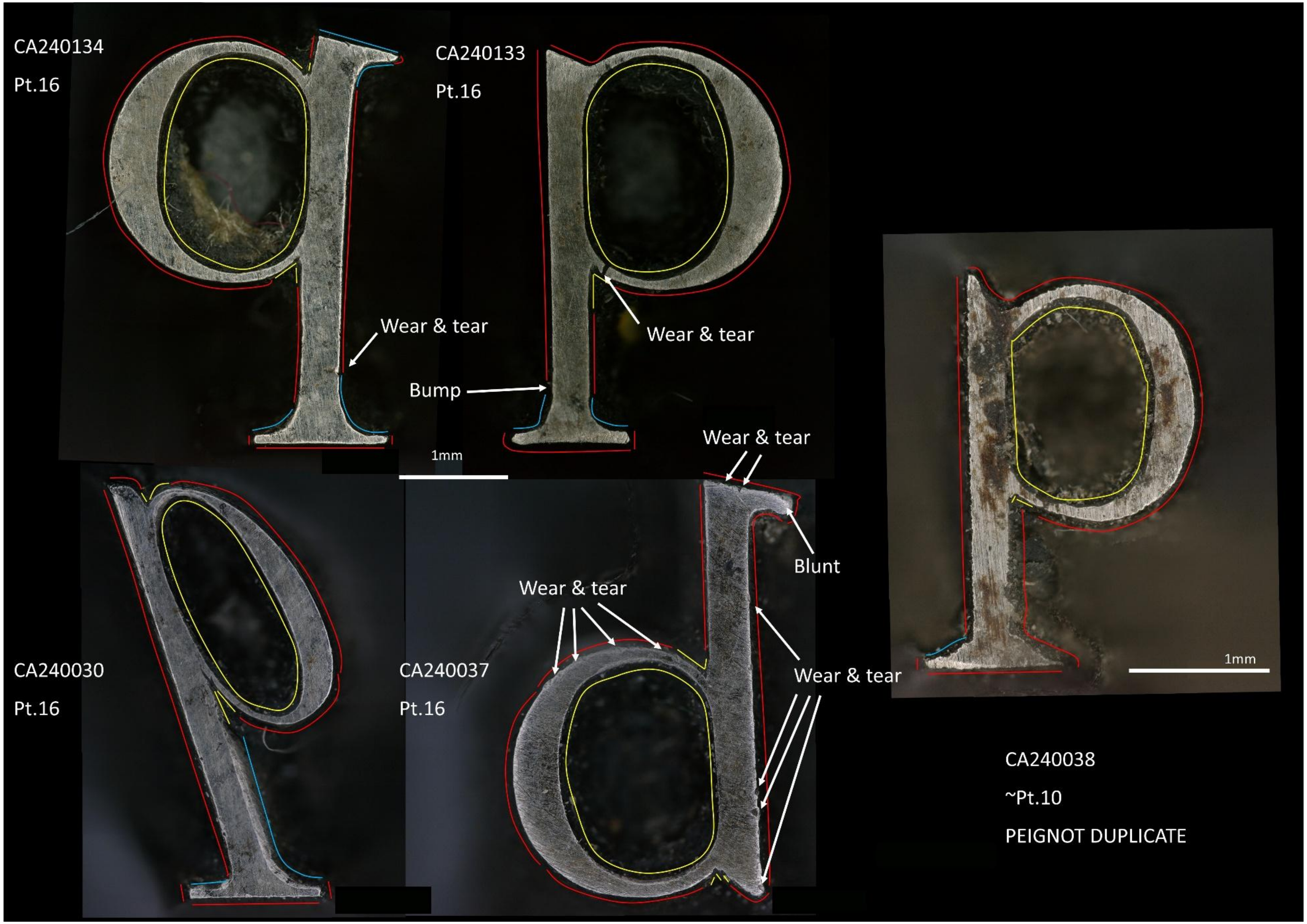

Supplementary Figure 7. Comparison of the techniques used to cut pt.16 roman p, q, and b, pt. 16 italic q, and pt.10 roman q. Red lines indicate areas of the punch cut by filing, yellow lines indicate areas of the punch cut by engraving, and blue lines indicate areas of the punch cut by a combination of filing and engraving.

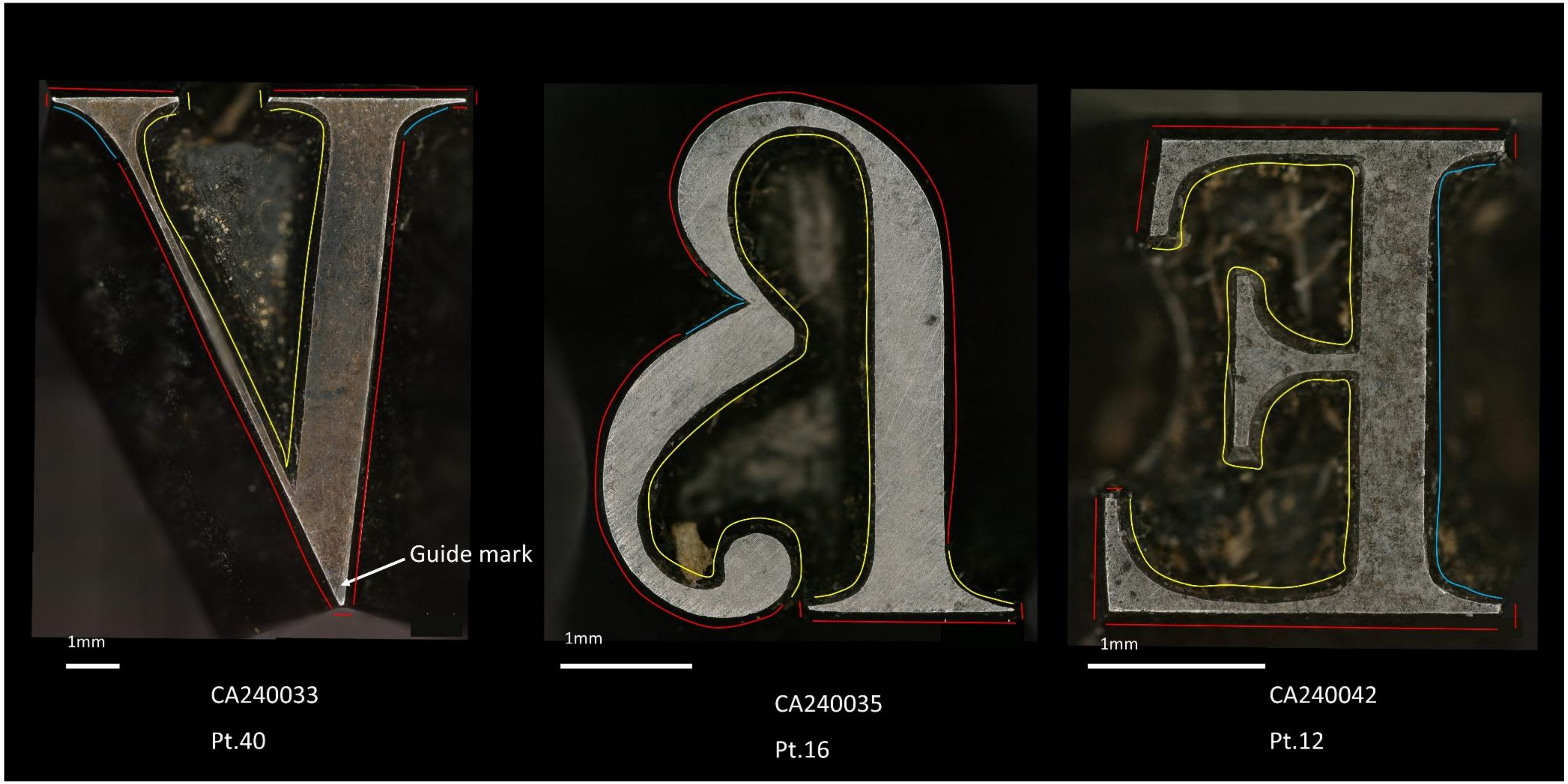

Supplementary Figure 8. Comparison of the techniques used to cut the modern punches pt.40 roman V, pt. 16 roman ß, and pt.12 roman E (step punch). Red lines indicate areas of the punch cut by filing, yellow lines indicate areas of the punch cut by engraving, and blue lines indicate areas of the punch cut by a combination of filing and engraving.

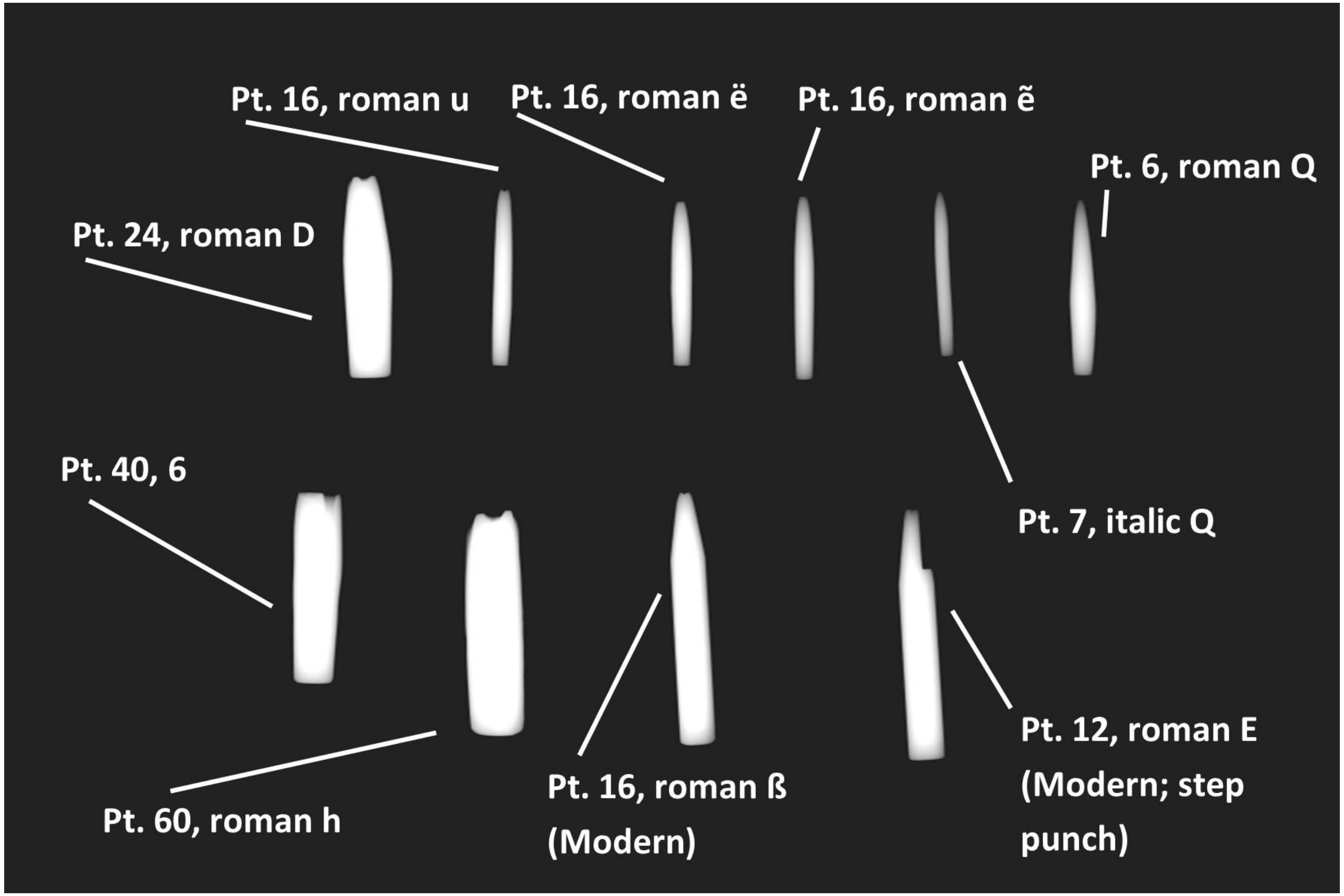

Supplementary Figure 9. Radiography of selected punches showing no welding lines.

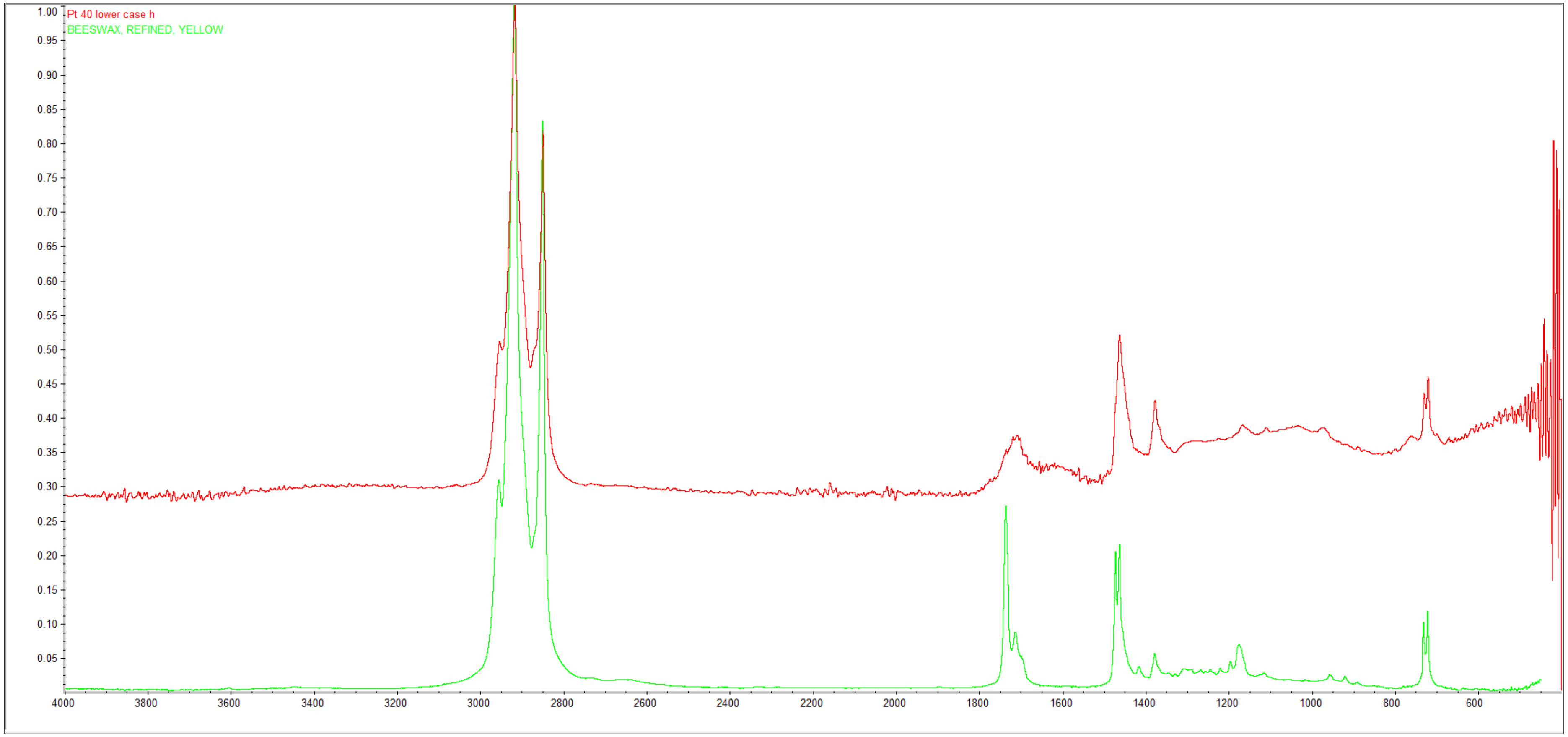

Supplementary Figure 10. FTIR spectra of bee wax (green) against the substance found in Pt.40, roman h (red).

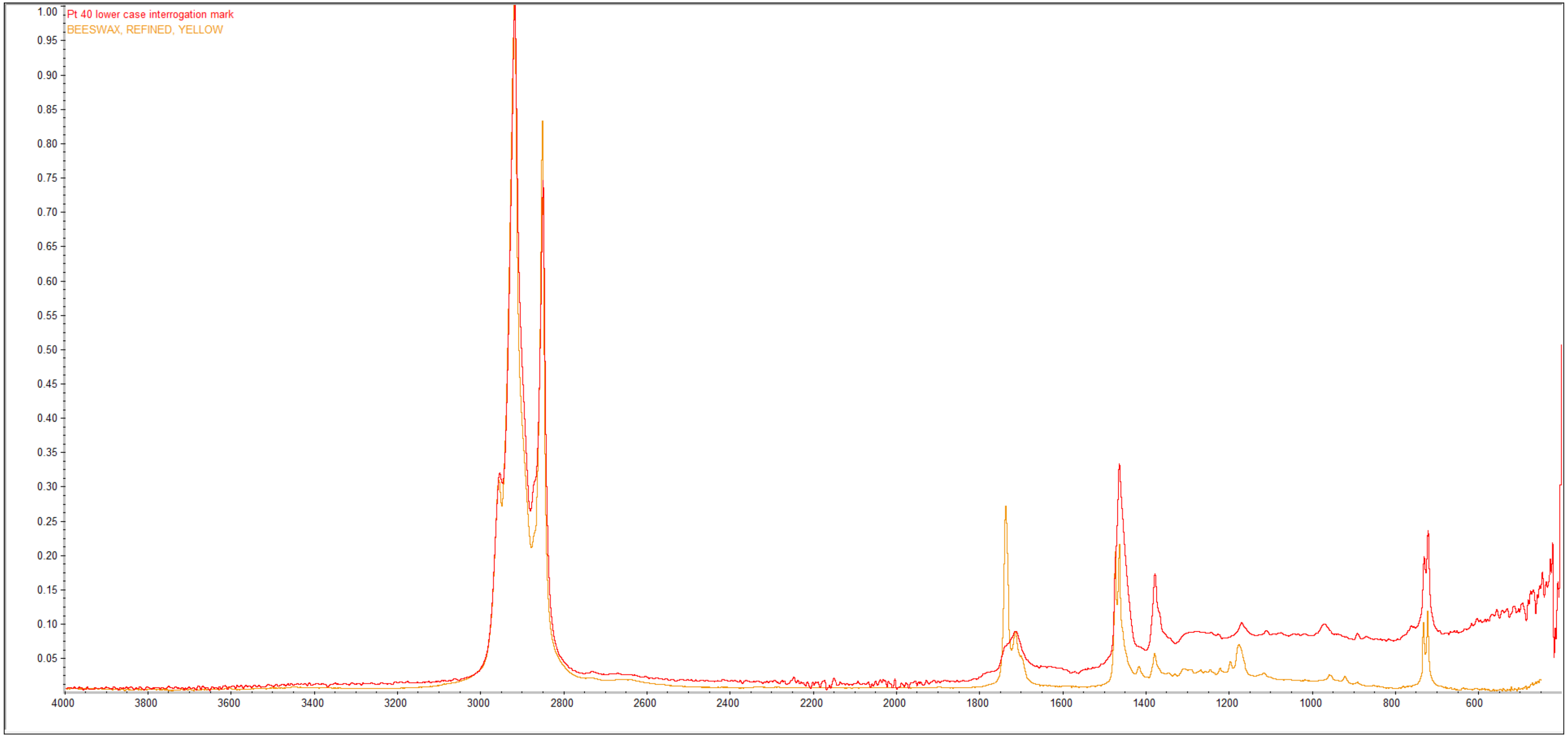

Supplementary Figure 11. FTIR spectra of bee wax (orange) against the substance found in Pt.40, roman ? (red).

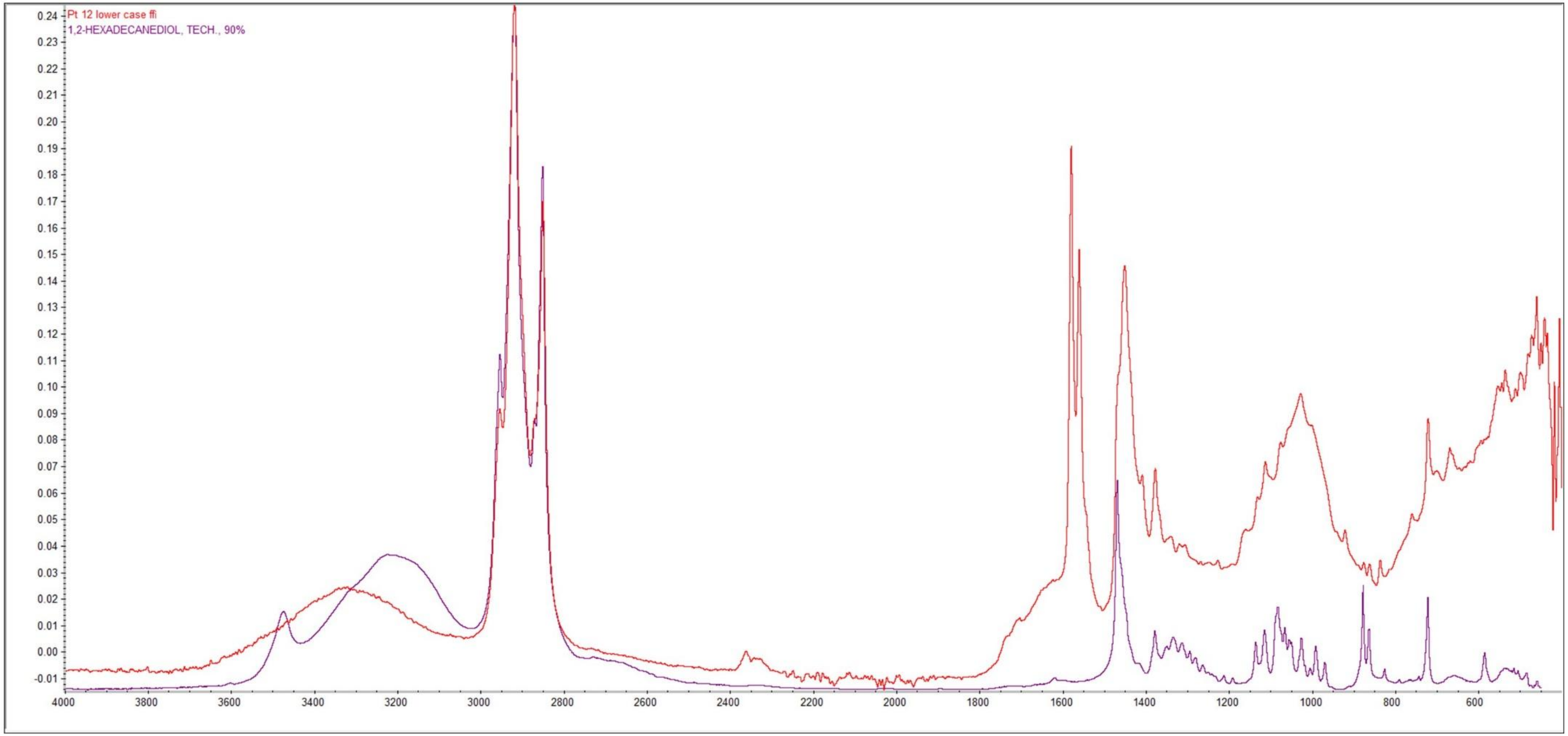

Supplementary Figure 12. FTIR spectra of hexadecanediol (purple) against the substance found in Pt.12, italic ffi (red).
