# Supplementary material for: The manufacture of the Baskerville typographic punches: the versatile chaîne opératoire of an 18th-century printing workshop
Source: NPJ Herit Sci. 2026 Apr 14;14(1):246. doi: 10.1038/s40494-026-02504-9 (PMC13079102; doi:10.1038/s40494-026-02504-9)
Supplement: Supplementary file 2 — Supplementary materials 2 [file 40494_2026_2504_MOESM2_ESM.zip › Supplementary materials 2/Supplementary Note 1. mCT movie captions.pdf]

Micro-CT movie captions

The manufacture of the Baskerville typographic punches: the versatile *chaîne opératoire* of an 18th century printing workshop

Julia Montes-Landa; Mark Box; Caroline Archer-Parré; Ann-Marie Carey; Maciej Pawlikowski; Marcos Martinón-Torres

Supplementary Movie 1. Micro-CT scan video of punch CA240035 (ß, roman, pt. 16).

Supplementary Movie 2. Micro-CT scan video of punch CA240036 (Q, roman, pt. 6).

Supplementary Movie 3. Micro-CT scan video of punch CA240039 (Q, italic, pt. 7).

Supplementary Movie 4. Micro-CT scan video of punch CA240041 (Q, roman, unknown pt.).

Supplementary Movie 5. Micro-CT scan video of modern punch CA240042 (E, roman, pt. 12).

Supplementary Movie 6. Micro-CT scan video of punch CA240111 (h, roman, pt.60).

Supplementary Movie 7. Micro-CT scan video of punch CA240113 (6, roman, pt.60).

Supplementary Movie 8. Micro-CT scan video of punch CA240119 (6, roman, pt. 40).

Supplementary Movie 9. Micro-CT scan video of punch CA240128 (ẽ, roman, pt. 16).

Supplementary Movie 10. Micro-CT scan video of punch CA240130 (u, roman, pt-16).

Supplementary Movie 11. Micro-CT scan video of punch CA240132 (ë, roman, pt.16).

Supplementary Movie 12. Micro-CT scan video of punch CA250014 (D, roman, pt.24).
